# Transcriptome Profiling of Powdery Mildew-Stressed ‘Yeniang No. 2’ Grapevine Reveals Differential Expression, Alternative Splicing, and the Identification of 1232 Annotated Novel Genes

**DOI:** 10.3390/metabo16030182

**Published:** 2026-03-09

**Authors:** Huan Yu, Essam Elatafi, Wen Liu, Rui Zhang, Basma Elhendawy, Shuyu Xie, Xiongjun Cao, Xianjin Bai, Qiumi Huang, Chunfen Jiang, Lei Wang, Jinggui Fang, Jiayu Han

**Affiliations:** 1 Grape and Wine Research Institute, Guangxi Academy of Agricultural Sciences, Nanning 530007, China; ankang1717@163.com (H.Y.); eelatafi@mans.edu.eg (E.E.); cxj4310@126.com (X.C.); b5629@126.com (X.B.); 19877140280@163.com (Q.H.); chunfen2026@163.com (C.J.); 2 College of Horticulture, Nanjing Agricultural University, Nanjing 210095, China; 2022204028@stu.njau.edu.cn (W.L.); zhangr@stu.njau.edu.cn (R.Z.); basmaelhendawy134@gmail.com (B.E.); 3 Department of Pomology, Faculty of Agriculture, Mansoura University, Mansoura 35516, Egypt; 4 Collaborative Innovation Center for Wine Industry Technology of Ningxia Helan Mountain Eastern Foothills, Yinchuan 750000, China; 13895186929@163.com

**Keywords:** grapevine, powdery mildew, RNA sequencing, transcriptome, differential gene expression, alternative splicing, functional enrichment, novel genes

## Abstract

**Background**: The global transcriptome reprogramming in grapevines in response to powdery mildew remains poorly understood, despite its economic implications, especially the new cultivars. **Methods**: Thus, this study aimed to elucidate these changes through RNA sequencing in ‘Yeniang No. 2’ grapevine leaves infected with powdery mildew compared to healthy ones. **Results**: A total of six samples were subjected to transcriptome sequencing, resulting in 36.85 Gb of clean data. A minimum of 5.89 Gb of clean data was generated for each sample, with at least 92.24% of the clean data attaining a quality score of Q30. Clean reads from each sample were aligned to the designated reference genome. The mapping ratio varied between 88.77% and 89.66%. The high-quality sequencing data revealed 1219 differentially expressed genes (DEGs), of which the infection upregulated 790 and downregulated 429. Functional enrichment analyses revealed a significant activation of key defense-related pathways. These included plant–pathogen interaction, phenylpropanoid and flavonoid biosynthesis for creating antimicrobial compounds, glutathione metabolism for reducing oxidative stress, and oxidative phosphorylation for enhanced energy production. This indicates a coordinated, multi-faceted defense strategy. The study also uncovered a complex layer of post-transcriptional regulation, identifying 1883 novel genes and 22,210 alternative splicing events, primarily skipped exons and intron retention. Key hub proteins identified within interaction networks, along with these splicing changes, underscore a sophisticated defense involving transcriptional reprogramming and metabolic shifts. **Conclusions**: The genes and molecular markers discovered are valuable resources for marker-assisted breeding. Leveraging these findings, particularly hub genes and favorable splice variants, can accelerate the development of new grapevine cultivars with durable resistance to powdery mildew.

## 1. Introduction

Grapevines (*Vitis* spp.) are a significant horticultural crop cultivated globally [1,2]. Most planted grapes presently belong to the species *Vitis vinifera*. They are extremely vulnerable to powdery mildew, a disease induced by the obligate biotrophic fungal pathogen *Erysiphe necator* Schw. *Erysiphe necator* originated in North America and disseminated to England and France in the 1840s, ultimately infiltrating all other viticultural regions globally [3]. The disease affects all green tissues during their growing seasons, significantly reducing productivity, fruit quality, and wine quality, compelling grape growers to provide frequent and expensive fungicide treatments [4]. Within the European Union, more than 80,000 tons of fungicides are utilized each year to manage powdery mildew, accounting for around 67% of the total fungicides employed across all crops, despite just 3.3% of agricultural land being allocated to grape cultivation. Powdery mildew accounts for approximately 20% of the expenses associated with wine grape cultivation in California. Excessive fungicide use is hazardous to both the environment and human health, while simultaneously heightening selection pressure on pathogen populations to adapt and develop greater fungicide resistance [5,6]. Consequently, developing grapevine varieties that are resistant to diseases presents a more sustainable and effective option while also being environmentally friendly.

Different grapevine species and cultivars have significantly different levels of resistance to powdery mildew. Although commercially prevalent *Vitis vinifera* varieties are often vulnerable, some wild species, including North American *Muscadinia rotundifolia* and *Vitis cinerea*; Chinese *Vitis* species such as *Vitis piasezkii*, *Vitis romanetii*, *Vitis quinquangularis*, and *Vitis pseudoreticulata*; and certain Central Asian accessions of *Vitis vinifera* ssp. sylvestris have been identified as having genetic resistance to powdery mildew [7,8,9]. Complex molecular networks, including various defense signaling pathways, secondary metabolite production, and cell wall modifications, primarily govern these differences in tolerance [4,5]. A prior work has shown that the R-gene *Resistance against Erysiphe necator 1 (Run1)*, mediated by a hypersensitive reaction, confers powdery mildew resistance in *Muscadinia rotundifolia* [10]. Fung et al. [11] utilized the *Vitis* GeneChip to report that 625 transcripts in *Vitis vinifera* and merely three transcripts in *Vitis aestivalis* were implicated in host responses to powdery mildew. Fekete et al. [12] found 25 genes in *Vitis vinifera* (cv. Cabernet Sauvignon) that were selectively elevated in response to *Erysiphe necator* and are susceptible to powdery mildew. Prior research offers significant insights into powdery mildew interactions within European and North American grape species.

Wine grape varieties exhibited differing degrees of sensitivity to powdery mildew due to their descent from *Vitis vinifera* L., a species that evolved without exposure to this fungus [13]. Conversely, the Chinese wild *Vitis* species co-evolved with *Erysiphe necator* and exhibited varying degrees of powdery mildew resistance, rendering these species a significant resource for investigating powdery mildew resistance in grapes. The Chinese wild *Vitis quinquangularis* Rehd. has potentially enduring, non-race-specific resistance to powdery mildew among wild *Vitis* species [9]. Genes conferring disease resistance have been effectively transferred from wild species of Chinese *Vitis* to *Vitis vinifera*.

The mechanisms of powdery mildew resistance in the Chinese wild species *Vitis pseudoreticulata* and *Vitis quinquangularis* were examined with a suppression subtractive hybridization cDNA library derived from *Erysiphe necator*-infected leaves [8]. Xu et al. [14] developed a cDNA library from *Vitis pseudoreticulata* infected with powdery mildew and discovered the expression of five sequence tags associated with powdery mildew protection. Investigations into gene expression profiling related to *Vitis pseudoreticulata* resistance have revealed the expression of *pathogenesis-related (PR)* genes, including *PR10*, a transcription factor potentially involved in conferring resistance to powdery mildew in grapevines [15]. Additional genes may significantly contribute to powdery mildew resistance, including *VpWRKY1*, *VpWRKY2*, *ethylene response factor*, *VpWRKY3*, *VpNAC1*, *ring finger protein VpRFP1* and its promoter, *heat shock transcription factor VpHsf1*, *retinoblastoma-related proteins*, *stilbene synthase (STS)*, *glyoxal oxidase*, and *aldehyde dehydrogenases* [16,17]. Zhang et al. [18] employed the *Vitis* microarray to examine gene expression in *Vitis pseudoreticulata* and identified the expression of 11,906 out of the 16,602 genes included on the microarray. Therefore, comprehending the genetic and molecular foundations of disease resistance and pinpointing the essential genes responsible for resistance in resilient germplasm should yield significant insights for employing molecular breeding to create novel grape varieties that demonstrate powdery mildew resistance while maintaining superior fruit quality. Consequently, the cultivar ‘Yeniang No. 2,’ derived from wild Chinese species, represents an underexplored genetic resource. It exhibits broad-spectrum disease resilience, including documented resistance to downy mildew [19,20]. Crucially, our field observations indicate that ‘Yeniang No. 2’ also possesses strong resistance to powdery mildew (*Erysiphe necator*). However, unlike its better-understood agronomic traits, the specific transcriptional mechanisms and key genes conferring this powdery mildew defense have not yet been identified.

In this study, we performed an extensive transcriptome analysis to delineate the molecular response of the ‘Yeniang No. 2’ grapevine to powdery mildew infection. Our primary objectives were to: (1) identify differentially expressed genes (DEGs) that are significantly up- or down-regulated following pathogen challenge; (2) discover novel, previously unannotated genes and analyze differential alternative splicing events associated with the defense response; and (3) perform functional enrichment analyses using GO, KEGG, and GSEA to elucidate the key biological pathways and protein interaction networks involved in powdery mildew resistance. This research seeks to delineate the transcriptional landscape of the grapevine–powdery mildew interaction, thereby offering a significant repository of candidate genes and pathways for subsequent functional investigations and the advancement of resistant cultivars via molecular breeding.

## 2. Materials and Methods

### 2.1. Plant Material, Growth Conditions, and Treatments

The experiment was carried out in a vineyard located at the Institute of Grapes and Wine in Nanning, Guangxi Province (22°36′39″ N, 108°13′51″ E). The grapevines utilized in the experiment were identified as ‘Yeniang No. 2.’ The cultivation occurred under a rain shelter, oriented in an east–west direction, with a spacing of 1.6 m between vines and 3.0 m between rows. A vertical canopy system alongside a conventional field management system was employed. The average irrigation rate was recorded at 21.6–36.0 L per vine per day during sunny conditions, while it was applied every other day under cloudy conditions. The cultivation and management of powdery mildew-infected ‘Yeniang No. 2’ were conducted using identical techniques to those employed for healthy ‘Yeniang No. 2,’ with the sole exception that no fungicide was utilized. Two experimental groups were utilized: healthy leaves (He) and leaves infected with powdery mildew (In). Each group consisted of three biological replicates, each of which comprised three grapevines. Throughout the winter fruit development period, spanning from late August to early January, the mean temperature recorded was 21.05 °C, accompanied by an average relative humidity of 81.40%. These climatic conditions were conducive to the proliferation of powdery mildew, particularly during the months of October to November, when the temperature reached 21.26 °C and relative humidity increased to 82.81%. The leaf samples were taken in autumn (18 November 2021), when the weather was drier with less rainfall, and powdery mildew was more severe. The leaves were collected from the third and fourth nodes, counting from the top. The samples were immediately frozen in liquid nitrogen after collection and stored at −80 °C for further analysis.

### 2.2. Total RNA Extraction and Transcriptome Sequencing

Grapevine leaf samples weighing 80 mg were ground in liquid nitrogen. Each sample underwent three biological replicates. The plant total RNA was extracted using the RNAprep Pure Plant Kit (Tiangen, Beijing, China) according to the instructions provided by the manufacturer. Subsequently, RNA concentration and purity were measured using NanoDrop 2000 (Thermo Fisher Scientific, Wilmington, DE, USA). RNA integrity was assessed using the RNA Nano 6000 Assay Kit of the Agilent Bioanalyzer 2100 system (Agilent Technologies, Santa Clara, CA, USA). Next, 1 μg RNA per sample was used as input material for the RNA sample preparations. Sequencing libraries were generated using Hieff NGS Ultima Dual-mode mRNA Library Prep Kit for Illumina (Yeasen Biotechnology, Co., Ltd., Shanghai, China) following manufacturer’s recommendations, and index codes were added to attribute sequences to each sample. mRNA was purified from total RNA using poly-T oligo-attached magnetic beads. Fragmentation was carried out using divalent cations under elevated temperature in NEBNext First Strand Synthesis Reaction Buffer (5X). First-strand cDNA was synthesized using random hexamer primer and M-MuLV Reverse Transcriptase. Second-strand cDNA synthesis was subsequently performed using DNA Polymerase I and RNase H. Remaining overhangs were converted into blunt ends via exonuclease/polymerase activities. After adenylation of 3’ ends of DNA fragments, NEBNext adaptors with hairpin loop structures were ligated to prepare for hybridization. To select cDNA fragments of preferentially 240 bp in length, the library fragments were purified with the AMPure XP system (Beckman Coulter, Beverly, MA, USA). Then 3 μL USER Enzyme (NEB, Ipswich, MA, USA) was used with size-selected, adaptor-ligated cDNA at 37 °C for 15 min followed by 5 min at 95 °C before PCR. Then PCR was performed with Phusion High-Fidelity DNA polymerase, Universal PCR primers, and Index (X) Primer. The PCR products were purified (AMPure XP system), and library quality was assessed on the Agilent Bioanalyzer 2100 system. At last, the clustering of the index-coded samples was performed on a cBot Cluster Generation System using TruSeq PE Cluster Kit v4-cBot-HS according to the manufacturer’s instructions. After cluster generation, the library preparations were sequenced on an Illumina NovaSeq6000 platform, and paired-end reads were generated. Following the library quality assessment, sequencing was commissioned to Shenzhen BGI Genomics Technology Co., Ltd. (Shenzhen, China) utilizing the DNBSEQ platform.

### 2.3. Quality Control and Sequencing Data Analysis

Raw reads underwent quality correction to yield valid sequences (clean reads). At the same time, Quality Score (Q) Q20, Q30, GC-content, and the sequence duplication level of the clean data were calculated [21]. For subsequent analysis, a proprietary reference genome of *Vitis adenoclada* (version Customer_v2) was used. This genome assembly was provided by Beijing Biomarker Biotechnology Co., Ltd., Beijing, China and is not publicly accessible but is available from the corresponding author upon reasonable request. Clean reads were aligned to this reference using HISAT2 [22]. StringTie [23] was applied to assemble the mapped reads by using Hisat2 alignment results. The quality of the RNA libraries was comprehensively evaluated prior to sequencing. We assessed RNA degradation and fragmentation randomness by examining the distribution of mapped reads across the genome. The library’s length dispersion was confirmed by analyzing the insert size distribution. Furthermore, the sufficiency of library volume was validated by generating a saturation curve, which correlated the number of genes identified with a specific expression accuracy to an increasing number of sampled mapped reads. Gene expression levels were quantified using FPKM (fragments per kilobase per million fragments mapped) by StringTie using the maximum flow algorithm [24]. The equation for FPKM is FPKM = cDNA Fragments/Mapped Fragments (Millions) ∗ Transcript Length (kb), where cDNA Fragments represents the number of PE reads mapped to the specific transcript; Mapped Fragments (Millions) is the number of all mapped reads, which is counted as 10^6^; and Transcript Length (kb) is the length of the transcript in units of 10^3^ b.

Differential expression analysis of two groups was performed using the DESeq2 [25] with three biological replicates. The criteria for identifying differentially expressed genes (DEGs) were set as a Fold Change (FC) of ≥2 and a False Discovery Rate (FDR) of <0.05. The *p*-values obtained were adjusted employing the Benjamini and Hochberg method to control the false discovery rate (FDR) [26]. Gene ontology (GO) enrichment analysis of DEGs was implemented by the GOseq R package based on Wallenius’ non-central hypergeometric distribution by using the clusterProfiler package according to Young et al. [27], which can adjust for gene length bias in DEGs. KOBAS digital library and clusterProfiler (v4.4.4) software were used to analyze the enrichment of DEGs in the KEGG (kyoto encyclopedia of genes and genomes) pathways [28] in the KEGG database. Gene set enrichment analysis (GSEA) was processed for all genes based on expression level. Gene sets of KEGG pathway and GO terms on biological process, cellular component, and molecular function were employed as gene sets of interest. Genes from each group were used as a background gene set. Enriched gene sets were identified with a *p*-value < 0.001 and FDR < 0.05 [29]. The sequences of the DEGs were blasted (blastx) to the genome of a related species (the protein–protein interaction (PPI) of which exists in the STRING database: http://string-db.org/ accessed on 2 November 2022) to get the predicted PPI of these DEGs.

The protein–protein interaction (PPI) network was constructed and analyzed using RStudio (v2025.09.2+418) with the tidyverse (v2.0.0), tidygraph (v1.3.0), and ggraph (v2.2.0) packages. The initial dataset, comprising a list of protein interactions with confidence scores and a corresponding node attribute file, was rigorously preprocessed to ensure data quality. Interactions with missing source or target identifiers were removed. To deal with duplicate entries where one protein pair had more than one interaction score, only the interaction with the highest score was kept for each unique pair. The network was treated as undirected. This filtering and cleaning procedure resulted in a final, high-confidence network of 1117 unique interactions among 427 distinct proteins (nodes). Topological properties of the network were calculated using functions from the tidygraph package. The degree centrality of each node was computed to quantify its number of connections. “Hub genes” were defined as the nodes within the top 10th percentile of the network’s degree distribution. To identify densely interconnected functional modules, a community detection analysis was performed using the Louvain algorithm, implemented via the group_louvain function. The algorithm was weighted by the Score of each interaction, giving higher-confidence interactions greater influence on the resulting community structure. The final PPI network was visualized using the ggraph package. The Fruchterman–Reingold force-directed layout algorithm, weighted by the interaction Score, was applied to organize the network structure, pulling tightly linked nodes closer together. To enhance interpretability, visual aesthetics were mapped to key topological attributes: node size was made proportional to its degree, and node color was assigned based on community membership. Edge width and transparency were mapped to the interaction score to highlight high-confidence connections. To maintain clarity, only the identified hub genes were labeled.

To investigate differential alternative splicing (AS), we analyzed RNA-seq data using the replicate multivariate analysis of transcript splicing (rMATS) pipeline [30]. We estimated the number of AS events in each sample separately. The analysis focused on five major types of AS events: skipped exon (SE), alternative 5’ splice site (A5SS), alternative 3’ splice site (A3SS), mutually exclusive exons (MXE), and retained intron (RI). For each event, the proportion of inclusion isoforms was quantified as the Percent Spliced In (PSI, or ψ) value, calculated from the counts of reads mapping to splice junctions. A likelihood-ratio test was conducted to evaluate the statistical significance of the disparity in PSI values between the two experimental groups. To account for multiple hypotheses testing, the resulting *p*-values were corrected using the Benjamini–Hochberg method to generate an FDR. Significant differential splicing events were defined as those meeting the following criteria: an FDR < 0.05 and an absolute change in PSI (|Δψ|) > 0.0001 [31].

The DEGs sets were used as candidate genes, and transcription factors (TFs) for plants were predicted using the software iTAK (v1.0) [32]. To identify genes with differential exon usage (DEU) between experimental conditions, we employed the DEXSeq package [32]. This method models exon-level read counts with a negative binomial distribution and utilizes a generalized linear model (GLM) to assess whether the relative usage of an exon, in proportion to the overall expression of its parent gene, differs significantly across sample groups. An FDR threshold of <0.05 was used to define statistically significant differential exon usage.

To validation the transcriptome results, ten DEGs were chosen for qPCR analysis utilizing the ChamQ Universal SYBR qPCR Master Mix Kit (Vazyme, Nanjing, China) and a Bio-Rad CFX-connected real-time system. The reaction conditions included pre-denaturation at 95 °C for 3 min, denaturation at 95 °C for 10 s, and annealing at 55 °C for 30 s. The final two steps were executed for 40 cycles. The data were analyzed using the 2^−∆∆CT^ technique [33], with *VvActin* serving as the internal reference gene. Primers were identified and developed utilizing the NCBI website (https://www.ncbi.nlm.nih.gov/) and Primer3 Plus software (v3.2.0) (Supplementary Table S1).

### 2.4. Statical Analysis

Analysis was performed using the statistical function prompt in R (v3.6.1). Unit variance was measured before unsupervised PCA. HCA results for samples were presented as dendrogram heatmaps [34]. Supplementary File S1 lists the software versions and their primary parameters along with a list of databases used in this study.

## 3. Results

### 3.1. Data Quality

Statistical analysis of clean sequencing data for all six samples (P1-He-A/B/C and P2-In-A/B/C) of ‘Yeniang No. 2’ grapevine confirmed the high quality of the run, which generated a total of 36.85 Gb of clean data (Supplementary Table S2). Each sample yielded 19.66 to 21.12 million clean reads, providing sufficient sequencing depth. Data quality was consistently high, demonstrated by stable GC content (45.88–46.55%) and excellent base call accuracy, with the percentage of bases exceeding a Phred score of 30% (≥Q30) consistently above 92.24%.

The quality of the RNA sequencing data, detailed in Supplementary Figures S1 and S2, was further assessed by aligning reads to the reference genome. The alignment resulted in a high overall mapping rate of 88.77% to 89.66% (Supplementary Table S3). The majority of these reads mapped uniquely (84.07–85.37%), with a low percentage of multi-mapping reads (3.70–4.81%), and no significant strand-specific bias was detected. Reads were uniformly distributed across all linkage groups without bias (Supplementary Figure S3), and a high proportion originated from exon regions (86.40–88.45%), confirming the successful capture of the expressed transcriptome (Supplementary Figure S4).

Three key quality control metrics ensured the reliability of the data. First, the uniform and symmetrical distribution of reads across normalized gene bodies indicated successful and random mRNA fragmentation (Supplementary Figure S5). Second, library insert sizes were consistent across all samples, showing a primary peak at approximately 320 bp, which confirmed the robustness of the preparation protocol (Supplementary Figure S6). Finally, sequencing saturation analysis demonstrated that the sequencing depth was sufficient for the reliable and accurate quantification of most expressed genes (Supplementary Figure S7).

### 3.2. Alternative Splicing Prediction

**Figure 1:**
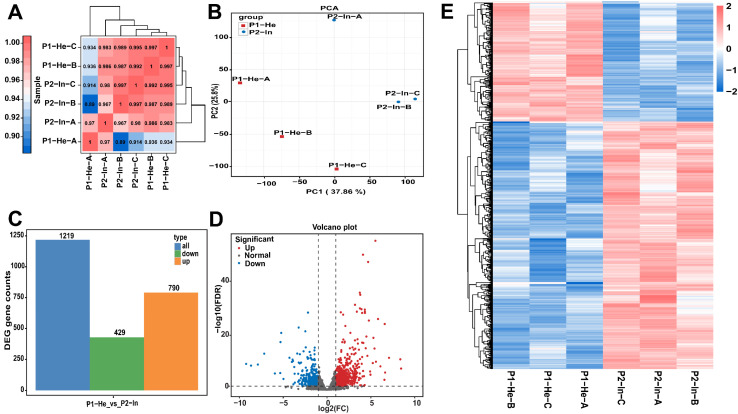
Transcriptome analysis and differential gene expression in grapevine leaves in response to powdery mildew infection. All analyses were performed on RNA-Seq data from healthy control (P1-He, *n* = 3) and powdery mildew-infected (P2-In, *n* = 3) leaves of grapevine ‘Yeniang No. 2’. (**A**) A heatmap of the Pearson correlation matrix, demonstrating high reproducibility among biological replicates. (**B**) Principal component analysis (PCA) plot showing distinct clustering of healthy and infected samples based on global gene expression profiles. (**C**) Statistical histogram of differential genes. (**D**) Volcano plot highlighting differentially expressed genes (DEGs) based on log_2_ fold change and statistical significance. (**E**) Hierarchical clustering heatmap of DEGs, showing distinct expression patterns between the two conditions.

To characterize the landscape of AS, RNA-Seq data from all samples were analyzed using the ASprofile pipeline [21]. Following transcript assembly with StringTie [23], a comprehensive identification and classification of AS events was performed. Events were categorized into 12 distinct types as shown in Supplementary Table S4. The frequency of each AS event type was quantified across all six samples (P1-He-A/B/C and P2-In-A/B/C), and the results are presented in Supplementary Figure S8A. A consistent distribution pattern was observed across all biological replicates and conditions. The most predominant forms of alternative splicing were Alternative 5’ first exon (TSS) and Alternative 3’ last exon (TTS), with the number of events for each type consistently exceeding 15,000 in every sample (Figure 1A). This suggests that variability in transcription initiation and termination is a major source of transcript diversity. Following TSS and TTS, Alternative Exon ends (AE) was the next most abundant category, with several thousand events detected per sample. Other common but less frequent events included Intron Retention (IR) and Skipped Exon (SKIP). In contrast, more complex events such as Multi-exon SKIP (MSKIP), Multi-Intron Retention (MIR), and all approximate-boundary variants (prefixed with ‘X’) were detected at significantly lower frequencies. The high degree of similarity in AS profiles across all samples indicates a stable and reproducible splicing landscape under the conditions studied. Additionally, the detailed catalog of all identified AS events was generated as shown in the Supplementary File S2.

### 3.3. Gene Structure Optimization and Novel Gene Analysis

To enhance the precision of the initial annotations derived from a reference genome, an analysis to optimize gene structure was conducted. This process involved correcting gene boundaries by examining mapped sequencing reads. In cases where continuous reads were found extending beyond the originally annotated gene boundaries, the untranslated regions (UTRs) were extended either upstream or downstream to refine the gene model. Through this analysis, a total of 2950 genes were successfully optimized. A detail of these genes was described in the Supplementary File S3, which compares the original annotated regions with the newly optimized regions.

To enhance the genomic annotation, a comprehensive analysis was conducted to identify and characterize novel genes. Novel transcripts were discovered by assembling mapped sequencing reads using StringTie and comparing the resulting assemblies against the existing reference genome annotation. Transcripts not corresponding to any previously annotated features were designated as novel. Following a filtering step to remove putative transcripts encoding peptides shorter than 50 amino acids and those comprising only a single exon, a total of 1883 novel genes were identified. The structural details of these genes, including their genomic coordinates, were compiled in Supplementary File S4.

To infer the biological roles of these newly identified genes, they were subjected to extensive functional annotation using a suite of bioinformatics tools and databases. Homology-based annotation was performed using DIAMOND [35] against the NR (Non-Redundant) [36], Swiss-Prot [37], COG (Clusters of Orthologous Groups) [38], KOG (eukaryotic Orthologous Groups) [39], and KEGG (Kyoto Encyclopedia of Genes and Genomes) [40] databases. Protein domains and families were identified by searching the Pfam [41] database using HMMER [42]. Additionally, Gene Ontology (GO) [43] terms were assigned using InterProScan [44], which integrates multiple signature-recognition methods (Supplementary File S5).

The annotation results were analyzed (Supplementary File S6). Of the novel genes analyzed, 1232 received a functional annotation in at least one of the queried databases. The TrEMBL database yielded the highest number of annotations (1198 genes), followed by GO (850 genes) and eggNOG (824 genes). Significant numbers of genes were also annotated against the KEGG (597), Pfam (585), Swiss-Prot (525), KOG (355), and COG (131) databases. Conversely, 1216 of the novel genes could not be assigned a function with the methods used, suggesting they may represent unique or lineage-specific genes requiring further investigation.

### 3.4. Expression and Differential Gene Analysis

Gene expression levels across six samples (P1-He-A/B/C and P2-In-A/B/C) were quantified as FPKM using StringTie (Supplementary File S7). Quality control analysis was performed to assess the comparability and integrity of the sequencing data. The distribution of gene expression values was found to be highly consistent across all samples, with each exhibiting a single, prominent peak, which indicates the absence of significant global biases. Box plot analysis of log10 (FPKM) values (Supplementary Figure S8B) confirmed this finding by showing that the medians and interquartile ranges of the samples were very similar. These results collectively demonstrate that the expression data were well normalized, with consistent magnitude and dispersion, rendering them suitable for subsequent differential expression analysis.

To evaluate the reproducibility among biological replicates and identify potential outliers [45], we performed correlation and clustering analyses. A Pearson correlation coefficient *r* was constructed and visualized as a heatmap (Figure 1C). The results demonstrated strong intra-group correlation, indicating high reproducibility among replicates within the same condition. For example, the correlation coefficients (*r*) between replicates in the P2-In group were 0.997, 0.980, and 0.967, while the coefficient between P1-He-B and P1-He-C was 0.997. Hierarchical clustering, illustrated by the dendrograms, segregated the samples into two distinct clusters corresponding precisely to the P1-He and P2-In experimental groups. This confirms that the primary source of variation in the dataset is the biological condition.

To further visualize the sample relationships, principal component analysis (PCA) was conducted on the FPKM data (Figure 1D). The PCA plot clearly and strongly separated the two experimental groups along the first principal component (PC1), which made up 37.86% of the total variance. The three P1-He replicates (red squares) clustered together, while the three P2-In replicates (blue circles) formed another well-defined cluster. This clear separation corroborates the heatmap analysis, providing strong evidence that the biological differences between the groups are the dominant feature in the gene expression data. Collectively, these quality control analyses confirm the high quality of the data and the consistency of the biological replicates, validating the dataset for subsequent analysis of differentially expressed genes.

To identify genes with significant expression changes between the P1-He and P2-In conditions, a differential expression analysis was performed using the DESeq2 package [25]. The analysis revealed a total of 1219 DEGs between the two groups. Of these, a majority of 790 genes were found to be significantly up-regulated in the P2-In group compared to the P1-He group. The remaining 429 genes were significantly down-regulated (Figure 1E). This indicates a substantial transcriptional response, with more genes being induced than repressed in the P2-In condition. The differential expression analysis output was shown in Supplementary File S8.

The overall distribution and statistical significance of the DEGs were visualized using a volcano plot (Figure 1D). The plot separates the significantly up-regulated genes (red points) and down-regulated genes (blue points) from the non-significant genes (grey points). The *y*-axis (−log10 (FDR)) shows that many of the identified DEGs have a high level of statistical confidence. The *x*-axis (log2(FC)) shows a wide range of fold changes, with some genes showing changes greater than 5-log2FC. This plot confirms that differential expression was detected across the full spectrum of gene expression levels, from lowly to highly expressed genes, and corroborates the set of significant up-regulated (red) and down-regulated (blue) genes.

To assess the overall expression patterns of the 1219 DEGs across all samples (Supplementary File S9), a hierarchical clustering analysis was performed. The resulting heatmap (Figure 1E) illustrates distinct transcriptional profiles between the P1-He and P2-In groups. The biological replicates within each condition (P1-He-A/B/C and P2-In-A/B/C) cluster tightly together, demonstrating high intra-group consistency and clear separation between the two conditions. The heatmap reveals two major gene clusters. The upper cluster consists of genes with consistently higher expression levels in the P2-In samples (shown in red) and lower expression in the P1-He samples (shown in blue), corresponding to the 790 up-regulated genes. Conversely, the lower cluster represents the 429 down-regulated genes, which exhibit lower expression in the P2-In samples and higher expression in the P1-He samples. This clear segregation of expression patterns confirms a robust and consistent transcriptional reprogramming between the P1-He and P2-In conditions.

### 3.5. Differential Gene Enrichment Analysis

To elucidate the biological functions of the 1200 DEGs identified between the P1-He and P2-In groups, a comprehensive functional analysis was conducted. The majority of DEGs were successfully annotated against multiple public databases (Supplementary File S10). The results shows that a vast majority of the DEGs received functional annotations. Specifically, 1198 DEGs were annotated in the non-redundant (NR) protein database, 1063 in the Pfam database, 1034 in the eggNOG database, 1008 in the gene ontology (GO) database, 967 in the swiss-prot database, and 877 were mapped to pathways in the kyoto encyclopedia of genes and genomes (KEGG) database. Furthermore, 458 DEGs were classified into the clusters of orthologous groups (COG) database, enabling a broad functional characterization.

**Figure 2:**
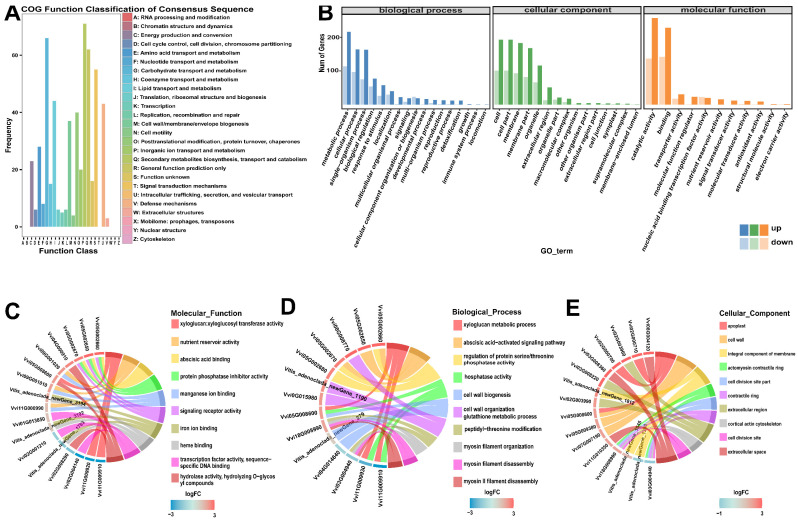
Functional annotation and enrichment analysis of differentially expressed genes (DEGs). DEGs were identified by comparing healthy control (P1-He, *n* = 3) and powdery mildew-infected (P2-In, *n* = 3) leaves of grapevine ‘Yeniang No. 2’. (**A**) Functional classification of DEGs based on Clusters of Orthologous Groups (COG) categories. (**B**) Gene Ontology (GO) classification of DEGs into the three main domains: Biological Process, Molecular Function, and Cellular Component. Chord diagrams visualizing the results of GO enrichment analysis for the most significant terms within the (**C**) biological process, (**D**) cellular component, and (**E**) molecular function domains, respectively, showing the relationships between GO terms and their associated DEGs.

Functional classification using the COG database revealed that the most highly represented categories were related to secondary metabolite and carbohydrate metabolism (Figure 2A; Supplementary File S11). Similarly, broad gene ontology (GO) classification showed that DEGs were most abundant in “metabolic process,” “cellular process,” “cell,” “membrane,” “catalytic activity,” and “binding” (Figure 2B; Supplementary File S12). Additionally, the top 10 enrichment GO terms were selected to draw an enrichment string graph, and the results were as shown in Figure 2C–E. 

**Figure 3:**
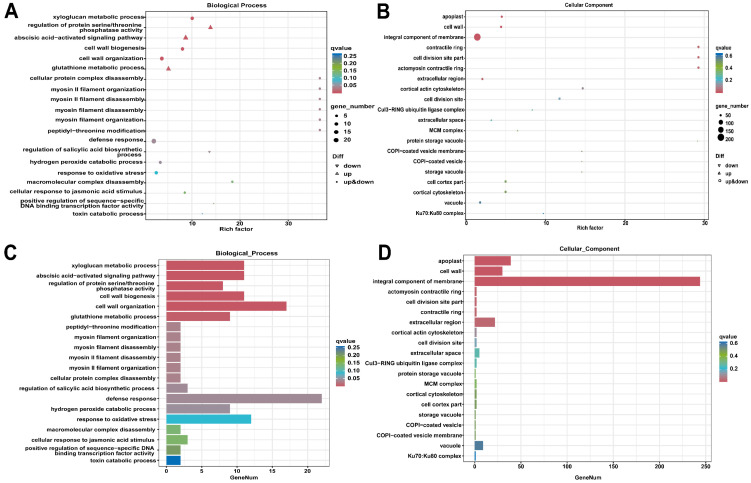
Gene ontology (GO) enrichment analysis was conducted on the differentially expressed genes (DEGs). DEGs were identified by comparing healthy control (P1-He, *n* = 3) and powdery mildew-infected (P2-In, *n* = 3) grapevine ‘Yeniang No. 2’ leaves. Bubble plots of the top enriched GO terms for the (**A**) biological process and (**B**) cellular component domains. Bar plots showing the number of DEGs in the most significantly enriched GO terms within the (**C**) biological process and (**D**) cellular component domains.

**Figure 4:**
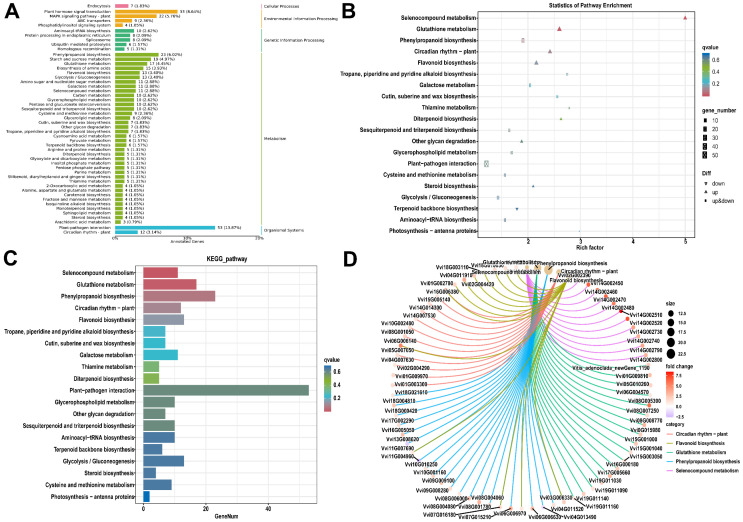
The Kyoto Encyclopedia of Genes and Genomes (KEGG) pathway analysis of differentially expressed genes (DEGs) in response to powdery mildew infection. DEGs were identified by comparing healthy control (P1-He, *n* = 3) and powdery mildew-infected (P2-In, *n* = 3) grapevine ‘Yeniang No. 2’ leaves. The KEGG database was used for pathway analysis. (**A**) Overview of KEGG pathway classifications, showing the distribution of DEGs across major biological categories. (**B**) A bubble plot of the top 20 most significantly enriched KEGG pathways. (**C**) A bar plot shows the number of DEGs assigned to the most enriched KEGG pathways. (**D**) A network visualization illustrating the relationships between significantly enriched KEGG pathways and their shared DEGs.

Subsequent GO and KEGG pathway enrichment analyses identified statistically over-represented functional categories and pathways (Figure 3, Figure 4, Figures S9, S13 and S14). Key enriched pathways included “Plant–pathogen interaction,” “Plant hormone signal transduction,” “Phenylpropanoid biosynthesis,” and “Starch and sucrose metabolism,” indicating that DEGs are primarily involved in plant defense, signal transduction, and metabolic regulation.

**Figure 5:**
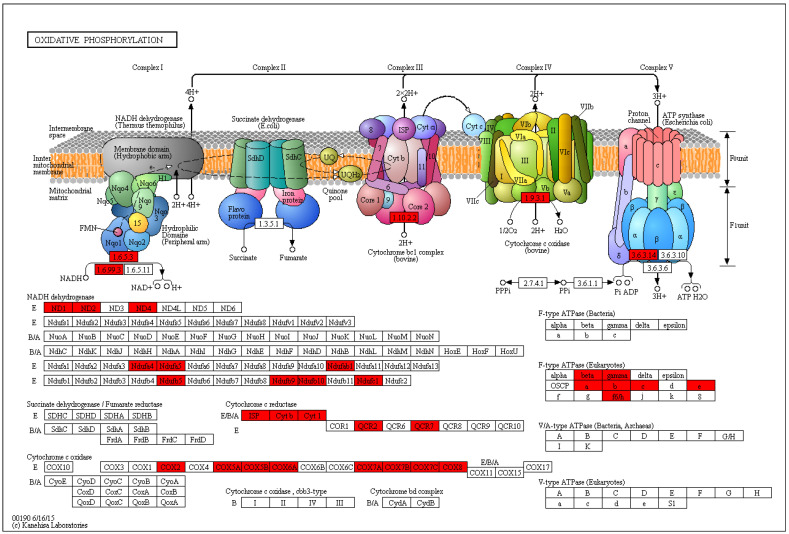
A Kyoto Encyclopedia of Genes and Genomes (KEGG) map of the biochemical and metabolic pathways shows differentially expressed genes. This KEGG pathway map illustrates the transcriptional response to powdery mildew infection in grapevine ‘Yeniang No. 2’ leaves. Differentially expressed genes (DEGs) identified between healthy control and infected samples were mapped onto the pathway. Enzymes, identified by their EC numbers, are color-coded based on the expression of their corresponding genes: red boxes indicate up-regulation.

A detailed examination of the “Oxidative Phosphorylation” pathway (Figure 5) reveals a striking and consistent pattern of gene regulation. The analysis showed a widespread upregulation of genes encoding subunits for all five major complexes of the mitochondrial electron transport chain. Specifically, multiple genes for NADH dehydrogenase (Complex I), such as ND1, ND2, ND3, ND4, ND4L, ND5, and ND6, were significantly upregulated. Likewise, parts of Succinate dehydrogenase (Complex II), the Cytochrome bc1 complex (Complex III), and Cytochrome c oxidase (Complex IV), such as COX1, COX2, and COX3, were also upregulated. This trend extended to ATP synthase (Complex V), where genes encoding subunits of the F-type ATPase were likewise upregulated. The pervasive upregulation across this entire pathway suggests a significant increase in mitochondrial respiratory activity and ATP production in the experimental condition relative to the control.

**Figure 6:**
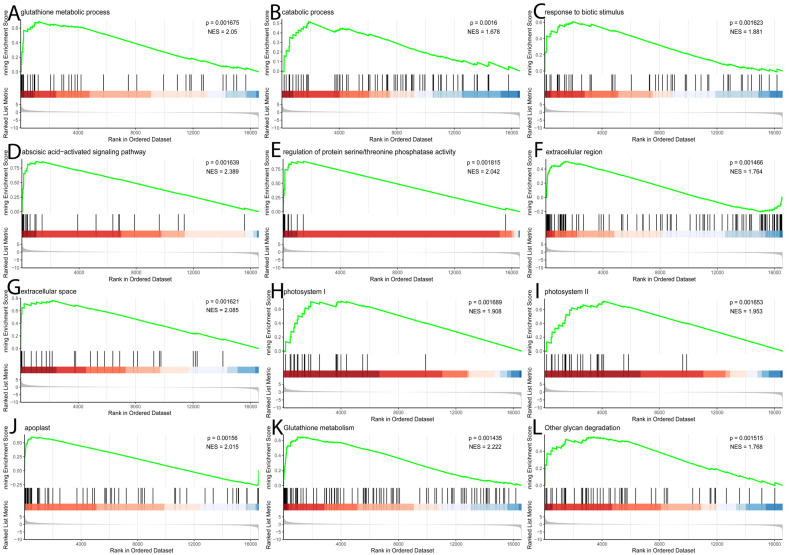
The Gene Set Enrichment Analysis (GSEA) plot displays the KEGG pathway along with the biological process, cellular component, and molecular function domains of the Gene Ontology (GO) gene set. The analysis compares gene expression profiles between healthy control (P1-He, *n* = 3) and powdery mildew-infected (P2-In, *n* = 3) grapevine ‘Yeniang No. 2’ leaves. The plot illustrates the distribution of a specific gene set across a list of all genes ranked by their log_2_ fold change (log2FC). The top panel shows the running enrichment score (ES) as a green curve, with its peak representing the final ES for the set. The middle panel is a barcode plot where each vertical line indicates the position of a gene from the gene set within the ranked list. The bottom panel displays the log2FC values for all ranked genes, with red indicating positive and blue indicating negative correlation. A positive ES signifies enrichment of the gene set among up-regulated genes, while a negative ES signifies enrichment among down-regulated genes.

Gene set enrichment analysis (GSEA) corroborated these findings, demonstrating significant enrichment of upregulated genes in pathways related to plant defense (“response to biotic stimulus”), secondary metabolism (“Phenylpropanoid biosynthesis”), signal transduction (“abscisic acid-activated signaling pathway”), and photosynthesis (Figure 6; Supplementary Figure S10, File S16). Significantly enriched biological processes included stress signaling (“abscisic acid-activated signaling pathway”) and the biosynthesis of defensive secondary metabolites (“Flavonoid biosynthesis,” “Phenylpropanoid biosynthesis”), along with robust oxidative stress management (“Glutathione metabolism”). This response was driven by key molecular functions, such as “manganese ion binding” and “UDP-glycosyltransferase activity.” At the cellular level, enrichment was concentrated in the “extracellular space” and “apoplast,” indicating active cell wall remodeling and secretion, while enrichment for “photosystem” suggests dynamic regulation of the photosynthetic machinery.

### 3.6. Differential Alternative Splicing and Differential Exon Analysis

The analysis of differential alternative splicing identified five primary types of AS events: skipped exon (SE), alternative 5’ splice site (A5SS), alternative 3’ splice site (A3SS), mutually exclusive exons (MXE), and retained intron (RI) (Supplementary Figure S11; Supplementary File S17). The analysis revealed a substantial number of differentials AS events, totaling 22,210 across all categories (Supplementary Table S4). Skipped exon (SE) was the most prevalent type of splicing event, with 15,139 instances identified. This was followed by intron retention (RI), with 2427 events. In order to visualize each differential splicing, the differential variable splicing results are visualized using rmats2sashimiplot software (v4.0.2). Taking an AS event of type SE as an example (Supplementary Figure S12).

DEU analysis was performed using the DEXSeq package (Supplementary File S18). DEU analysis provides insights into expression differences between sample groups at the exon level, enabling the identification of specific exons that influence the expression of functional genes. This approach, in conjunction with the study of alternative splicing events, facilitates a more detailed understanding of gene expression regulatory mechanisms. For example, Table 1 presents a selection of exons identified as having significant differential usage between the experimental groups. The analysis identified exons with both increased (positive log2(FC)) and decreased (negative log2(FC)) usage. For instance, exon E001 of the gene *Vad01G006980* exhibited a highly significant increase in usage (log2(FC) = 0.86, FDR = 7.70 × 10^−7^), while exon E002 of *Vad01G003820* showed significantly decreased usage (log2(FC) = −0.76, FDR = 0.029). All listed exons met the significance threshold (FDR < 0.05), indicating specific alternative splicing events that differ between the conditions.

Additionally, the Supplementary Figure S13 provides a detailed visualization of a significant DEU event identified in the gene ENSSSCG00000000886, comparing a “P2-In,” group (red lines) to a “P1-He” group (blue lines). The analysis revealed that exon E007, highlighted in purple, exhibits markedly reduced usage in the “P2-In” group. This finding is supported from multiple perspectives within the figure. The modeled expression estimates show a clear drop in the relative abundance of exon E007 in the “P2-In” group (Panel A), a trend that is confirmed by the lower normalized read counts across individual biological replicates (Panel C). Critically, after normalizing for overall gene expression differences, the splicing model (Panel D) demonstrates a sharp decrease in the relative inclusion rate of exon E007. This isolates the change as a specific splicing event rather than a consequence of lower gene-level transcription. Collectively, these visualizations provide compelling evidence for a condition-specific alternative splicing event, likely the skipping of exon E007, in the “P2-In” group.

### 3.7. Protein–Protein Interaction Network

**Figure 7:**
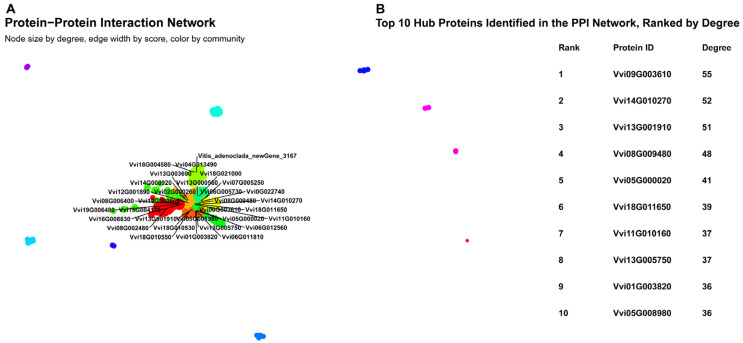
Protein–protein interaction (PPI) network of differentially expressed genes in grapevine. The network was constructed using genes differentially expressed between healthy control leaves and leaves infected with powdery mildew from the grapevine cultivar ‘Yeniang No. 2’. Each node represents a protein, and edges indicate interactions. Node size is proportional to its degree, with larger nodes indicating more connections. Gene colors correspond to network communities 1–17 as defined in the community_color_legend.csv file in Supplementary File S19.

Figure 7A shows a protein–protein interaction network. The overall topology reveals one large, densely interconnected component surrounded by several smaller, isolated subgraphs and individual nodes (Supplementary File S19). The main component is characterized by a high degree of connectivity, with numerous edges converging on central nodes. These larger, central nodes represent “hub” proteins, which are involved in a high number of interactions and are likely critical for the network’s integrity and function. A community detection analysis, visualized by color, indicates a modular structure. The main component is composed of multiple, closely interacting communities, primarily colored red, orange, yellow, and green (Supplementary File S19). This modularity suggests the presence of distinct functional modules or protein complexes within the larger network. The smaller, disconnected subgraphs also form their own communities (e.g., cyan, blue, and magenta), indicating groups of proteins that interact with each other but not with the main network component. Additionally, the table in Figure 7B gives a numerical summary of the network’s most important proteins. It shows the top 10 hub proteins, which were found and ranked based on their degree. The protein with the highest connectivity is *Vvi09G003610*, with a degree of 55. The degrees of the top 10 hub proteins range from 55 down to 36, highlighting the key players within the interaction network.

### 3.8. Validation of qPCR

To validate the accuracy of transcriptome sequencing, 10 DEGs were randomly chosen for qPCR investigation. The expression patterns of these genes corresponded with the transcriptome sequencing data (Supplementary Figure S14). The results unequivocally proved the trustworthiness and precision of the transcriptome sequencing data.

## 4. Discussion

This comprehensive transcriptome analysis demonstrated that the ‘Yeniang No. 2’ grapevine exhibited a significant transcriptional response to powdery mildew infection. A total of six samples were subjected to transcriptome sequencing, resulting in 36.85 Gb of clean data. A minimum of 5.89 Gb of clean data was generated for each sample, with at least 92.24% of the clean data attaining a quality score of Q30. Clean reads from each sample were aligned to the designated reference genome. The mapping ratio varied between 88.77% and 89.66%. Alternative splicing prediction, gene structure optimization, and novel gene discovery were conducted based on mapping results, leading to the identification of 1883 genes and the annotation of 1232 novel genes with putative functions. Furthermore, DEGs were identified using the criteria of Fold Change ≥ 2 and FDR < 0.01, resulting in a total of 1219 DEGs, predominantly upregulated (790 genes).

Our study demonstrates that high sequencing depth, stable GC content, and Q30 scores exceeding 92% reflect superior sequencing quality, consistent with established benchmarks for plant RNA-seq research. The consistent exon mapping rate (86.40–88.45%) and elevated unique mapping (84.07–85.37%) indicate effective library preparation and dependable transcriptome coverage. Technical performance is recognized as crucial for accurate quantification and reproducibility in RNA-seq-based research on plant stress [46,47,48]. The strong intra-group correlation, distinct group separation in PCA, and well-normalized FPKM distributions underscore significant biological repeatability and minimal technical noise. Previous RNA-seq studies on grapevine drought and salt stress have highlighted the critical role of correlation and clustering among replicates in differentiating genuine biological responses from artifacts [46,49]. Additionally, hierarchical clustering and volcano plot visualization demonstrate substantial DEGs discovery, a trend seen in *Vitis* investigations under drought or salinity where extensive transcriptome remodeling underpins adaptive responses. DEGs enrichment in plant–pathogen interaction, MAPK signaling, phenylpropanoid, flavonoid, and glutathione metabolism pathways matches significant molecular themes in grapevine and other crops under biotic and abiotic stressors. These pathways are linked to stress signaling, secondary metabolite production, and detoxification [50,51,52,53,54].

In our findings, according to the GO analysis, the most DEGs were found in broad functional categories such as “metabolic process,” “cellular process,” “cell,” “membrane,” “catalytic activity,” and “binding.” Enrichment analysis revealed that these broad categories, as well as more particular pathways linked to membrane systems, organelles, and enzymatic processes, are the most significantly over-represented and biologically relevant. Upregulated genes were primarily related with these enriched terms. A KEGG enrichment analysis indicated that the DEGs response was mostly linked to plant defense mechanisms and significant metabolic alterations. The most notably enhanced pathways were “Plant–pathogen interaction” and “MAPK signaling,” signifying a major involvement in defense responses. Moreover, a pervasive activation of genes throughout all complexes in the oxidative phosphorylation pathway indicated a substantial enhancement in mitochondrial energy generation. Significant enrichment was observed in key metabolic pathways for secondary metabolites (“Phenylpropanoid biosynthesis,” “Flavonoid biosynthesis”) and primary metabolism (“Starch and sucrose metabolism,” “Biosynthesis of amino acids”), highlighting a thorough reprogramming of both defense and metabolic processes. GSEA indicated a statistically significant and coordinated upregulation of genes associated with a comprehensive biotic stress response. This includes the activation of defense and secondary metabolite pathways, stress hormone signaling, oxidative stress management, and adaptive remodeling of photosynthetic and extracellular systems. In *Vitis* and other stress transcriptomics literature, functional annotation and KEGG/GO enrichment results showed metabolic adjustment (starch/sucrose, amino acids), hormone signaling, and antioxidant defense are involved. GSEA confirmed the induction of defense, signaling, photosynthetic repair, and oxidative stress responses, similar to grape, tomato, cucumber, and flax treated with stress combos or dynamic abiotic triggers [49,54,55,56,57,58,59].

Our results from a comprehensive analysis of differential alternative splicing (DAS) identified five primary types of alternative splicing (AS) events, totaling 22,210 significant AS events, predominantly consisting of skipped exon (SE) types (15,139). Additionally, differential exon usage analysis confirmed specific exons that exhibited altered splicing between the experimental conditions. The identification of extensive alternative splicing (AS), characterized by predominant TSS, TTS, and AE, along with numerous significant differential splicing events, particularly SE and RI, aligns with existing multi-cultivar grape RNA-seq analyses. These studies acknowledge AS a key factor in proteome plasticity under stress conditions. The prevalence of exon skipping and the stability of alternative splicing profiles across replicates have been emphasized in recent cross-stress studies involving grapevine and other crops, raising awareness about the importance of alternative splicing in adaptive gene regulation [47,58,60]. Furthermore, the identification of 1883 new genes and the development of gene models demonstrate the ongoing expansion of annotated grapevine transcriptomes in our study, where the recent *Vitis* investigations indicated that high-depth RNA-seq routinely identifies lineage-specific, stress-regulated new genes with potential roles in adaptation, prompting continued annotation improvements [46,59].

In our findings, the developed PPI network exhibited a highly centralized structure, primarily characterized by two principal hub proteins, Vvi09G003610 and Vvi14G010270. Their extensive connectedness and closely linked neighbors indicate that these hubs are crucial regulatory proteins essential to the functional interactions among the DEGs. Analyses of protein–protein interaction networks indicate that hub regulators align with recent co-expression and PPI studies in grapevine related to drought, heat, and pathogen responses. In these analyses, central nodes often correspond to transcription factors, kinases, and regulators of stress pathways [48,49,52,61]. In addition to using rMATS and DEXSeq to determine condition-specific alternative splicing and differential exon use, recent stress transcriptomics techniques have been developed to analyze post-transcriptional complexity. Condition-associated AS events support the idea that stress affects gene abundance and isoform variety, affecting plant plasticity and adaptation [58,60].

Altogether, the current results confirm and extend significant molecular trends identified in grapevine and broader plant transcriptomics. Specifically, stress induces extensive transcriptional reprogramming, alternative splicing, and the enrichment of defense, metabolic, and signaling networks. The integration of AS, DEU, network analysis, and novel gene discovery illustrates a complex, multi-layered regulatory response in *Vitis*, which is increasingly acknowledged as essential for resilience to both biotic and abiotic challenges [47,49,52,53].

## 5. Conclusions

This comprehensive transcriptome analysis revealed that the ‘Yeniang No. 2’ grapevine exhibited a significant transcriptional response to powdery mildew infection, identifying 1219 differentially expressed genes (DEGs), predominantly upregulated (790 genes). Functional enrichment analyses, including GO, KEGG, and GSEA, indicated significant activation of defense-related pathways, such as plant–pathogen interaction, phenylpropanoid and flavonoid biosynthesis, glutathione metabolism, and oxidative phosphorylation. This data indicates a systematic approach to the development of antimicrobial agents, reduction in oxidative stress, and enhancement of energy production. Our study identified 1883 unique genes and significant post-transcriptional regulation, revealing 22,210 distinct alternative splicing events, primarily consisting of skipped exon and intron retention, indicating a complex layer of gene expression control. A limitation of the current study, however, is that these alternative splicing events were identified computationally. Future experimental validation of these specific transcriptional variants, such as verifying skipped exons and intron retentions via RT-PCR, is required to confirm these in silico transcriptomic predictions. The identification of key hub proteins in protein interaction networks and the common exon-level alterations underscore a complex defense strategy involving transcriptional reprogramming, metabolic transitions, and advanced splicing processes. Consequently, the genes and molecular markers discovered here represent valuable resources for marker-assisted breeding. Developing functional markers based on favorable splice variants or expression polymorphisms of hub genes following their experimental validation could accelerate the development of new durable–resistant grapevine cultivars.

## Figures and Tables

**Table 1:** Significant differential exon usage events were identified in grapevine leaves upon infection with powdery mildew. The table shows a list of some exons with statistically significant changes in usage between healthy control and infected leaves of *Vitis vinifera* cv. ‘Yeniang No. 2’.

Gene ID	Exon ID	log2 (FC)	*p*-Value	FDR < 0.05
Vvi01G000440	E010	0.45	3.89936586901781× 10^−6^	0.0093
Vvi01G006980	E001	0.86	4.60421704722239× 10^−11^	7.69745683931335× 10^−7^
Vvi01G007020	E001	−0.27	5.1444472820316e× 10^−10^	5.33084775521721× 10^−6^
Vvi02G010110	E009	1.23	1.67971793480829× 10^−6^	0.0042
Vvi03G006200	E009	0.61	2.6730703629865× 10^−7^	0.001
Vvi04G000160	E007	−0.61	1.25989085152904× 10^−6^	0.0035
Vvi04G013390	E002	0.23	9.0380399016407× 10^−7^	0.0026
Vvi05G001490	E003	0.74	1.41709569359706× 10^−6^	0.0038
Vvi05G002690	E006	0.47	4.65762569471482× 10^−8^	0.00022

Note: Log2 (FC): log2 (Fold change); *p*-value: Significancy of difference; FDR: False discovery rate.

## Data Availability

Upon reasonable request, the corresponding authors can provide the raw transcriptome sequencing data presented in this study. Additionally, the data presented in this study is available within this manuscript and Supplementary Materials.

## Supplementary Materials

The following supporting information can be downloaded at: https://www.mdpi.com/article/10.3390/metabo16030182/s1, Figure S1: Distribution of base call error rates for raw sequencing data, Figure S2: Per base sequence content of raw sequencing reads, Figure S3: Genomic distribution and coverage depth of sequencing reads, Figure S4: Read mapping distribution across the reference genome for six grapevine samples, Figure S5: Assessment of read coverage uniformity across mRNA transcripts, Figure S6: Quality assessment of sequencing libraries based on insert size distribution, Figure S7: Sequencing saturation analysis of RNA-Seq libraries, Figure S8: The distribution of different types of alternative splicing events detected and Boxplots of FPKM values consistent expression distributions across samples, Figure S9: Bubble plots of the top enriched GO terms for the molecular function domain, Figure S10: The Gene Set Enrichment Analysis plot displays the KEGG pathway along with the molecular function domains of the GO gene set, Figure S11: Analysis of differential alternative splicing events between healthy and powdery mildew-infected grapevine leaves, Figure S12: Sashimi plot of a differential skipped exon event in grapevine ‘Ye Niang 2’, Figure S13: Visualization of differential exon usage for a representative gene in grapevine, Figure S14: Correlation heatmap of gene expression profiles for qPCR validation, Table S1: Primer pairs for qPCR analysis of 10 DEGs, Table S2: Evaluating the quality and summarizing RNA-sequencing libraries, Table S3: Alignment statistics of RNA-sequencing reads to the grapevine reference genome, Table S4: Summary of alternative splicing events in healthy and infected grapevine leaves, Table S5: Classification and quantification of differential alternative splicing events in response to powdery mildew infection, File S1: List of software and database, File S2: Catalog of all identified AS events, File S3: Gene structure optimization and novel gene analysis, File S4: The structural details of novel genes with their genomic coordinates, File S5: Complete GO annotation results from InterProScan, File S6: Summary of functional annotation results across all queried databases, File S7: Gene expression values (FPKM) for all samples, File S8: Complete list of differentially expressed genes, File S9: Expression data for all 1219 DEGs across six samples, File S10: Functional annotation results for all 1200 DEGs against multiple public databases, File S11: COG functional classification results for DEGs, File S12: Gene Ontology classification results for DEGs, File 13: Complete GO enrichment analysis for all DEGs, including biological process, cellular component, and molecular function domains with enrichment statistics, File S14: KEGG pathway enrichment analysis results, File S15: TopGO enrichment results for up-regulated and down-regulated DEGs across three domains, File S16: Gene set enrichment analysis results showing significantly enriched pathways among upregulated and downregulated genes, File S17: Differential alternative splicing events identified across all samples, including five AS types, File S18: Differential exon usage analysis results, File S19: Full network topology data including node and edge lists, connectivity metrics, hub protein identification, and community detection assignments with color codes.
