# Supplementary material for: Transcriptome Profiling of Powdery Mildew-Stressed ‘Yeniang No. 2’ Grapevine Reveals Differential Expression, Alternative Splicing, and the Identification of 1232 Annotated Novel Genes
Source: Metabolites. 2026 Mar 9;16(3):182. doi: 10.3390/metabo16030182 (PMC13027967; doi:10.3390/metabo16030182)
Supplement: Supplementary file 1 [file metabolites-16-00182-s001.zip › Supplementary Figures.pdf]

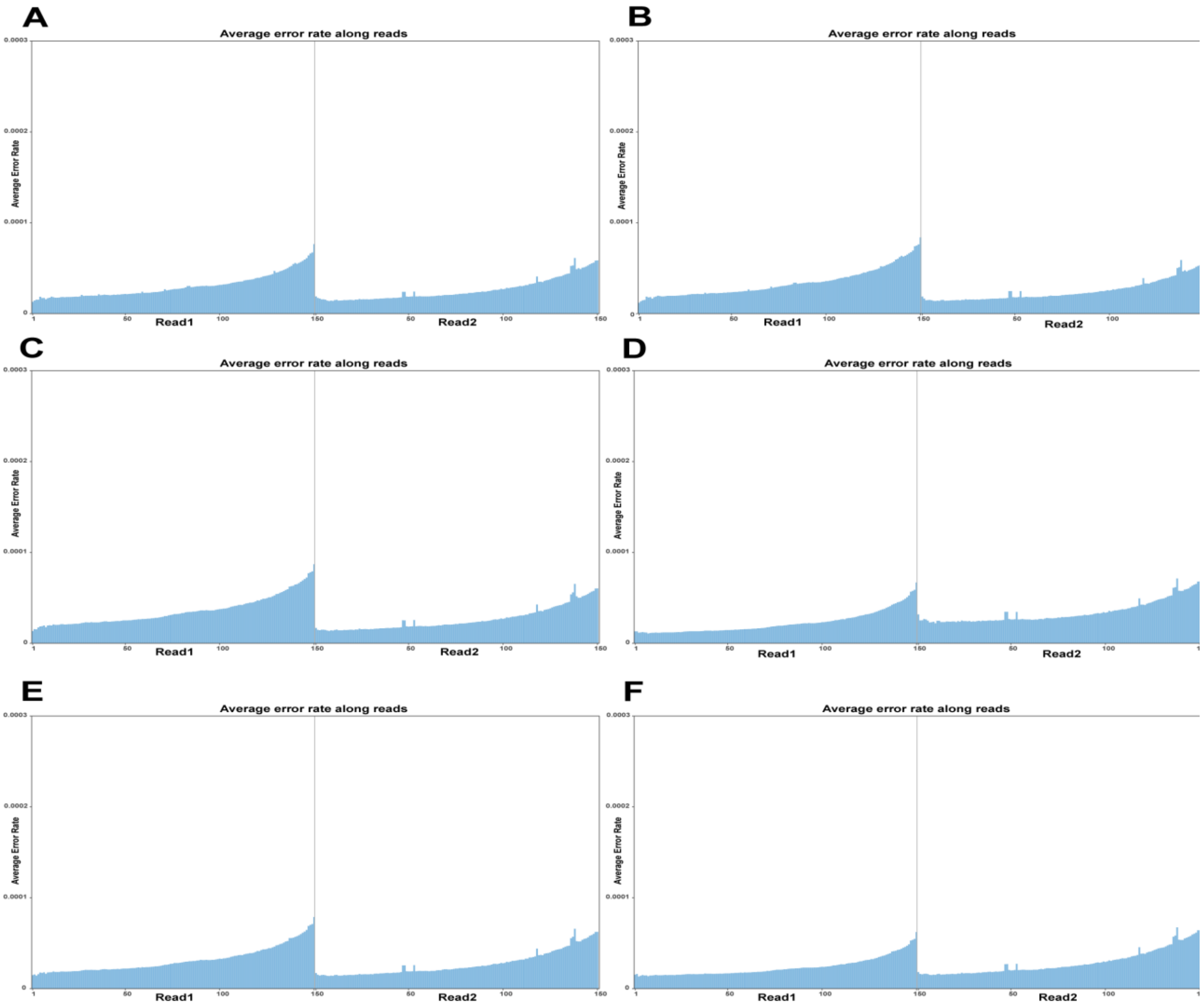

Supplementary Figure S1. Distribution of base call error rates for raw sequencing data. The six samples were from grapevine 'Ye Niang 2' and represent two conditions: healthy control leaves (P1-He-A, P1-He-B, P1-He-C; n=3) and leaves infected with powdery mildew (P2-In-A, P2-In-B, P2-In-C; n=3).

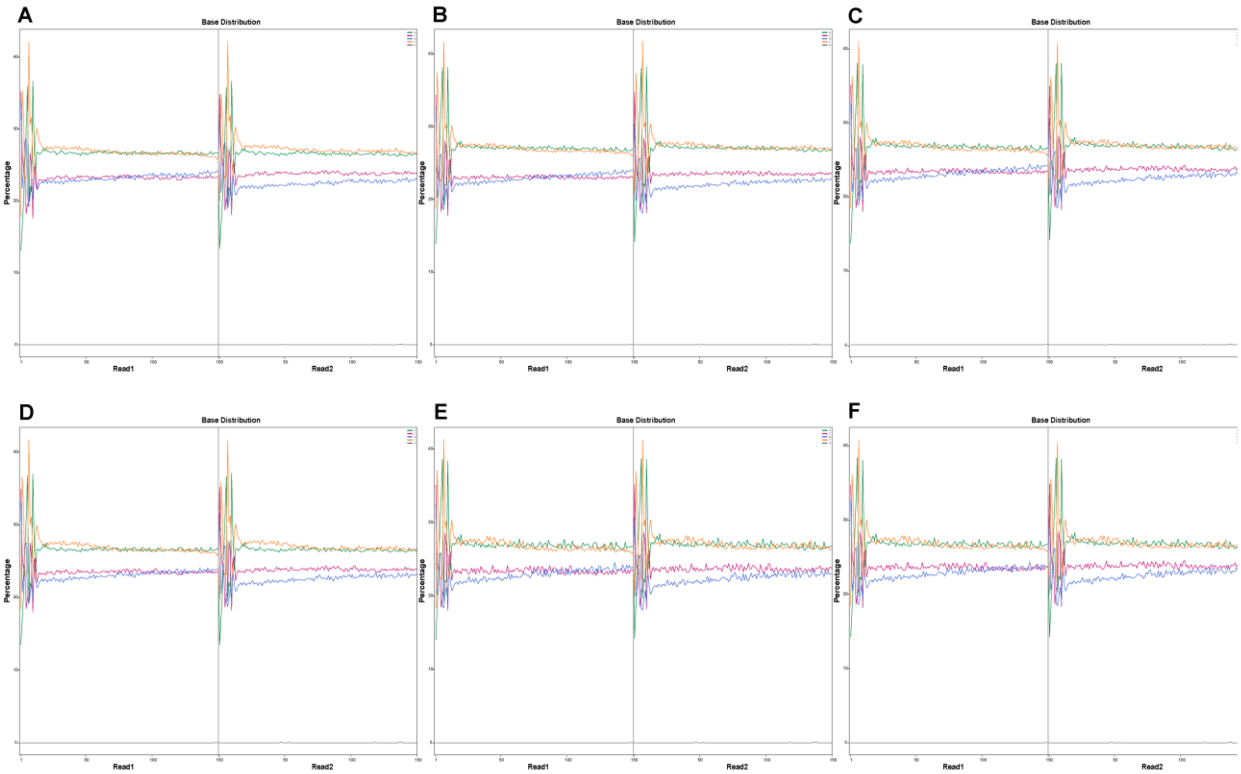

Supplementary Figure S2. Per base sequence content of raw sequencing reads. This plot displays the proportion of each nucleotide (A, T, C, G) at each position along the reads for healthy (P1-He) and powdery mildew-infected (P2-In) grapevine leaves 'Ye Niang 2', with three biological replicates per condition.

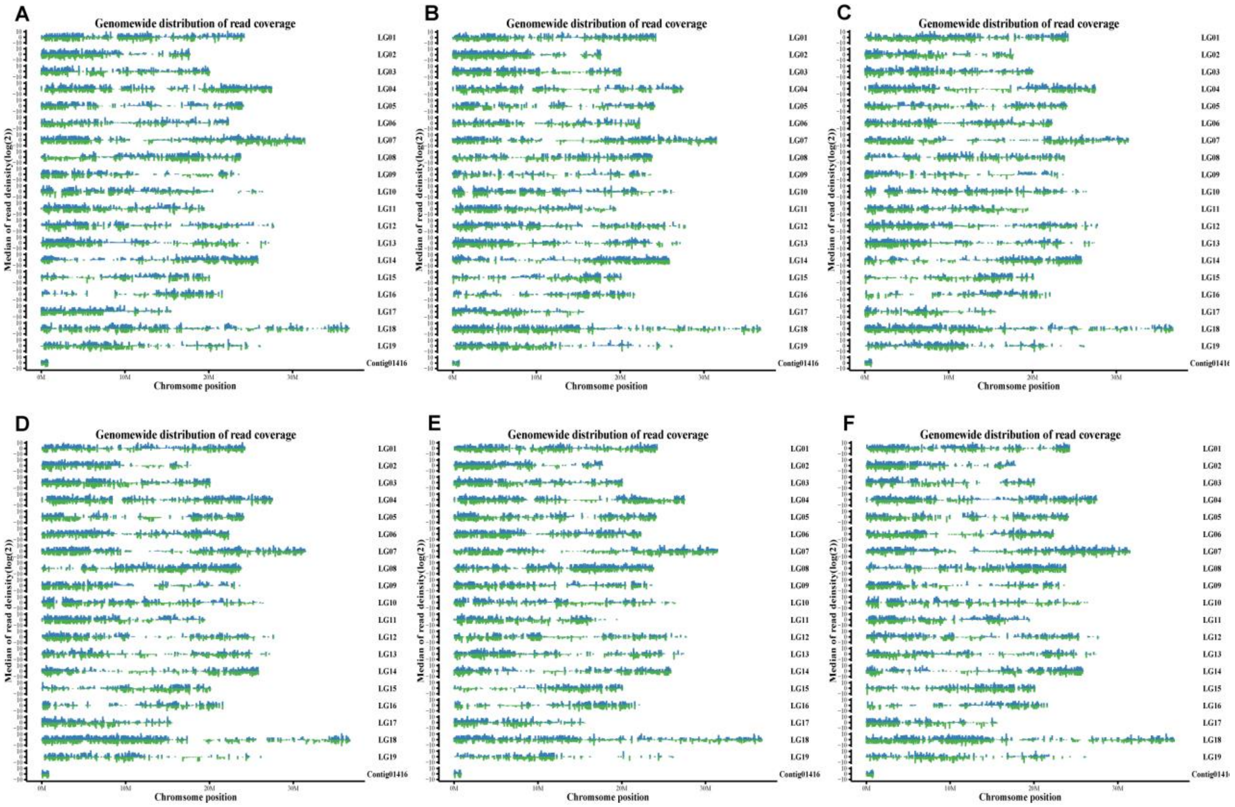

Supplementary Figure S3. Genomic distribution and coverage depth of sequencing reads. Reads were mapped to the reference genome for six grapevine 'Ye Niang 2' samples. The samples represent two conditions: healthy control leaves (P1-He-A, P1-He-B, P1-He-C) and leaves infected with powdery mildew (P2-In-A, P2-In-B, P2-In-C), each with three biological replicates (n=3).

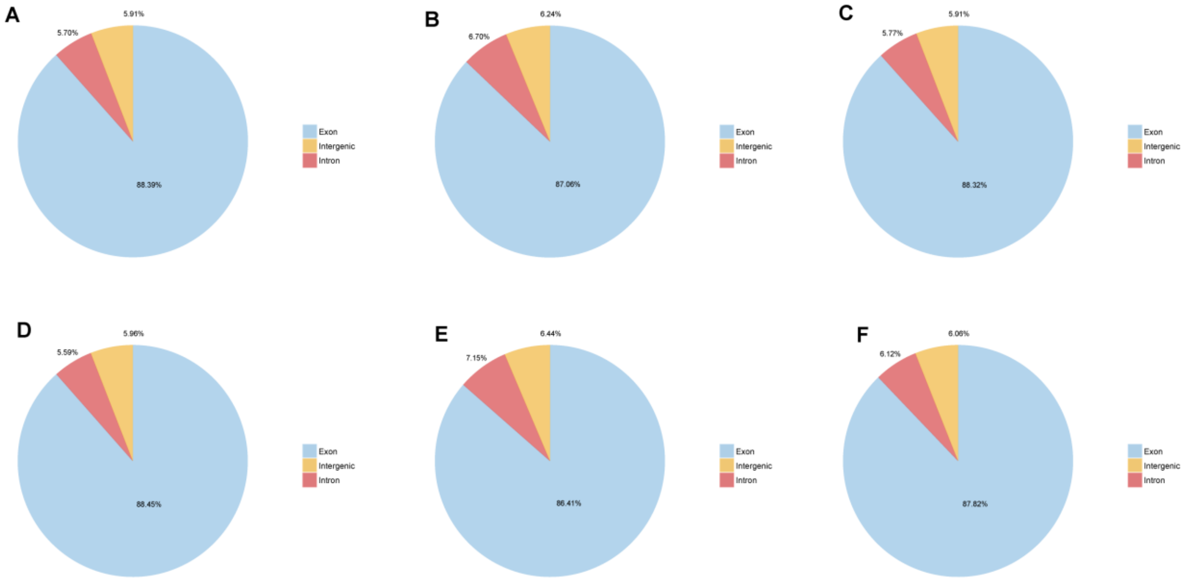

Supplementary Figure S4. Read mapping distribution across the reference genome for six grapevine samples. The analysis compares healthy control leaves (P1-He-A, P1-He-B, P1-He-C) with leaves infected by powdery mildew (P2-In-A, P2-In-B, P2-In-C) from the cultivar ‘Ye Niang 2’.

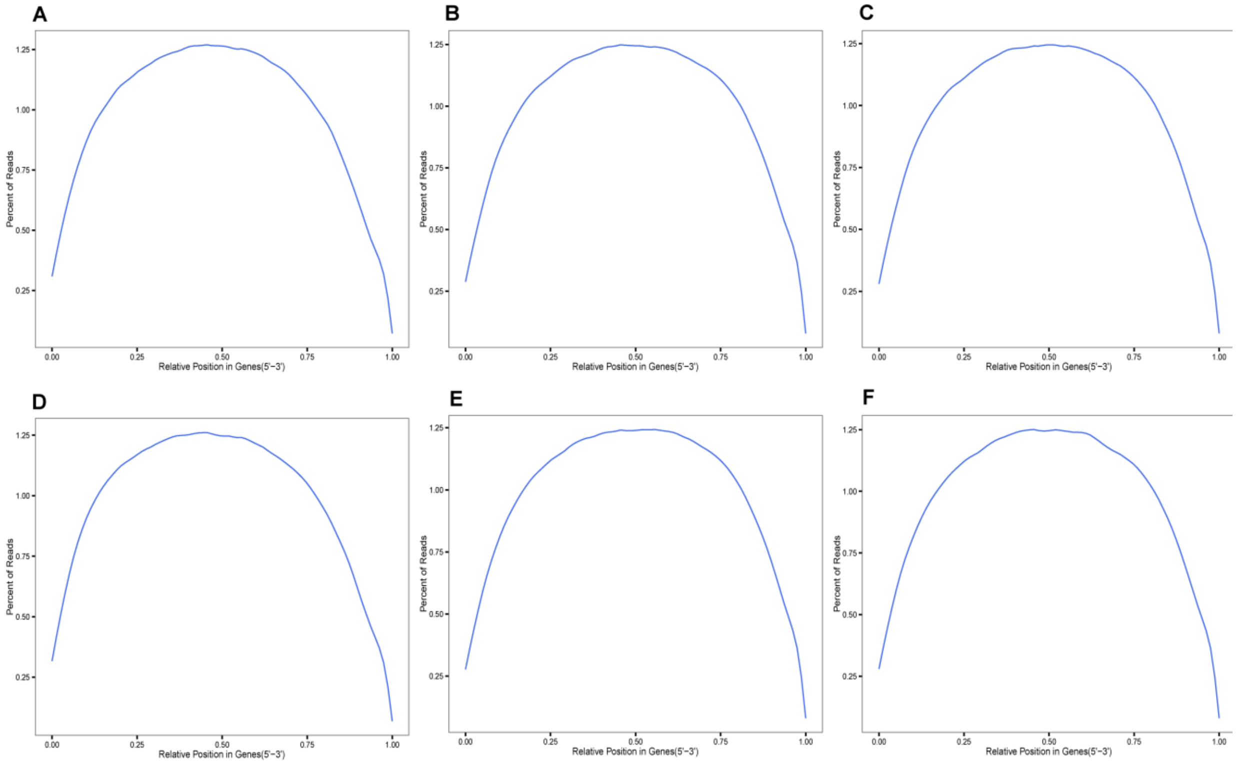

Supplementary Figure S5. Assessment of read coverage uniformity across mRNA transcripts. The distribution of sequencing reads was plotted along the relative length of transcribed gene bodies (from 5' to 3' UTRs). This analysis was performed on six samples of grapevine 'Ye Niang 2', representing two conditions: healthy control leaves (P1-He-A, P1-He-B, P1-He-C) and leaves infected with powdery mildew (P2-In-A, P2-In-B, P2-In-C).

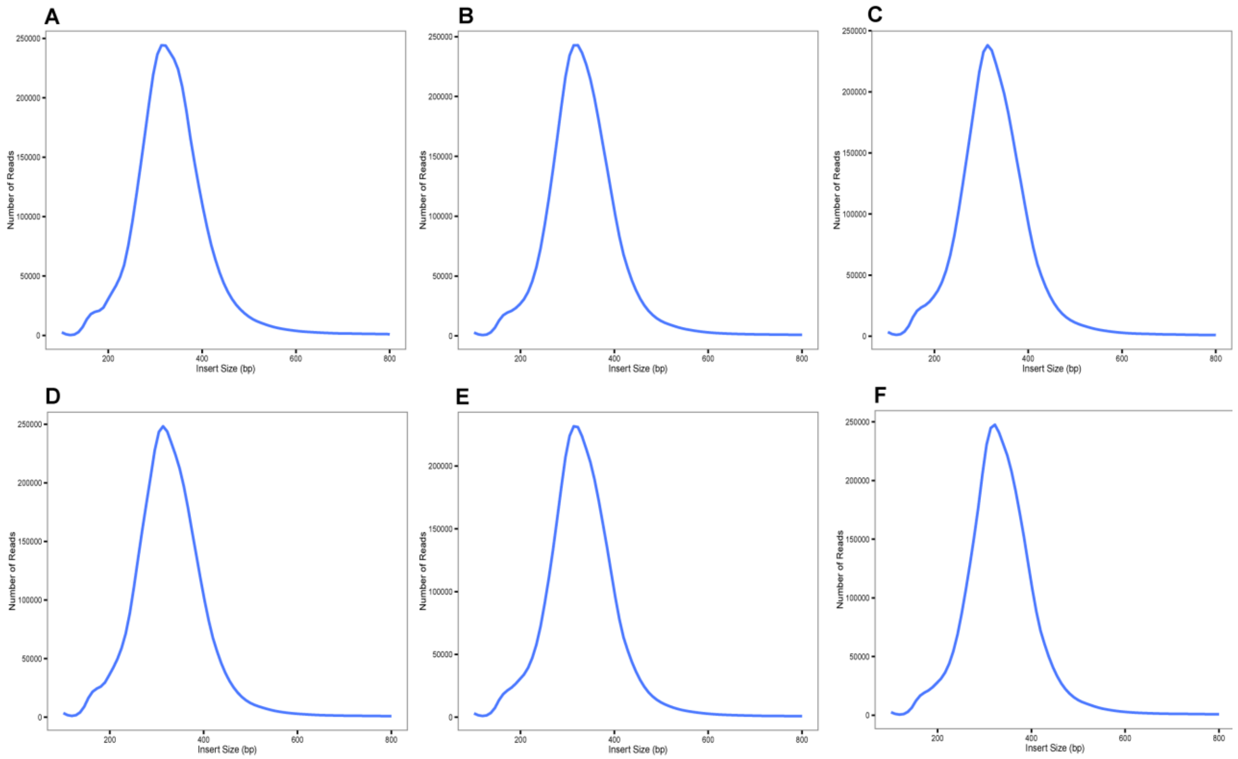

Supplementary Figure S6. Quality assessment of sequencing libraries based on insert size distribution. The plot displays the frequency of insert sizes, representing the length of the DNA/cDNA fragment between paired-end adapters. This analysis includes six libraries from grapevine 'Ye Niang 2', comprising three biological replicates each of healthy control (P1-He) and powdery mildew-infected (P2-In) conditions.

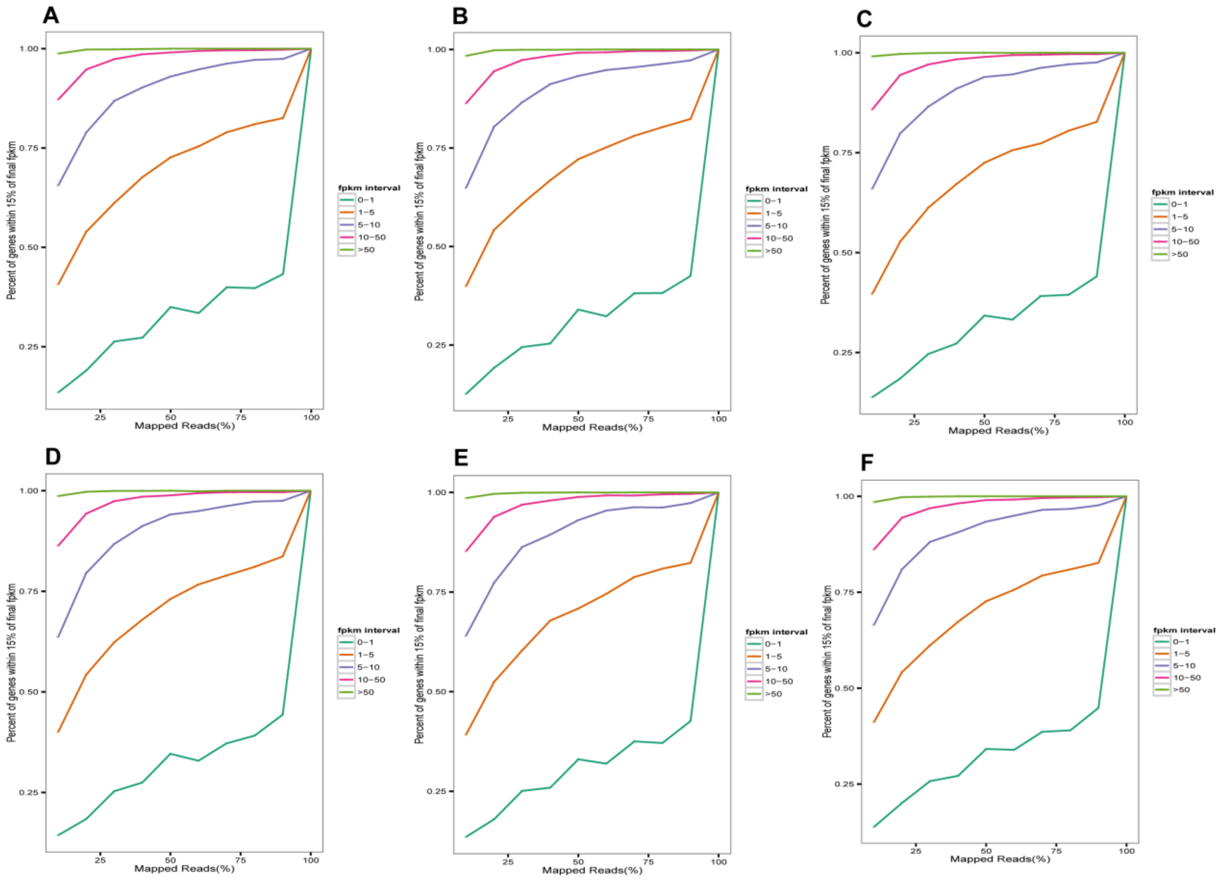

Supplementary Figure S7. Sequencing saturation analysis of RNA-Seq libraries. The plot shows the number of detected genes as a function of increasing sequencing depth for healthy control (P1-He; n=3) and powdery mildew-infected (P2-In; n=3) leaf samples from grapevine ‘Ye Niang 2’. The plateauing curves for all samples indicate that the sequencing depth was sufficient to capture most expressed genes in the transcriptome.

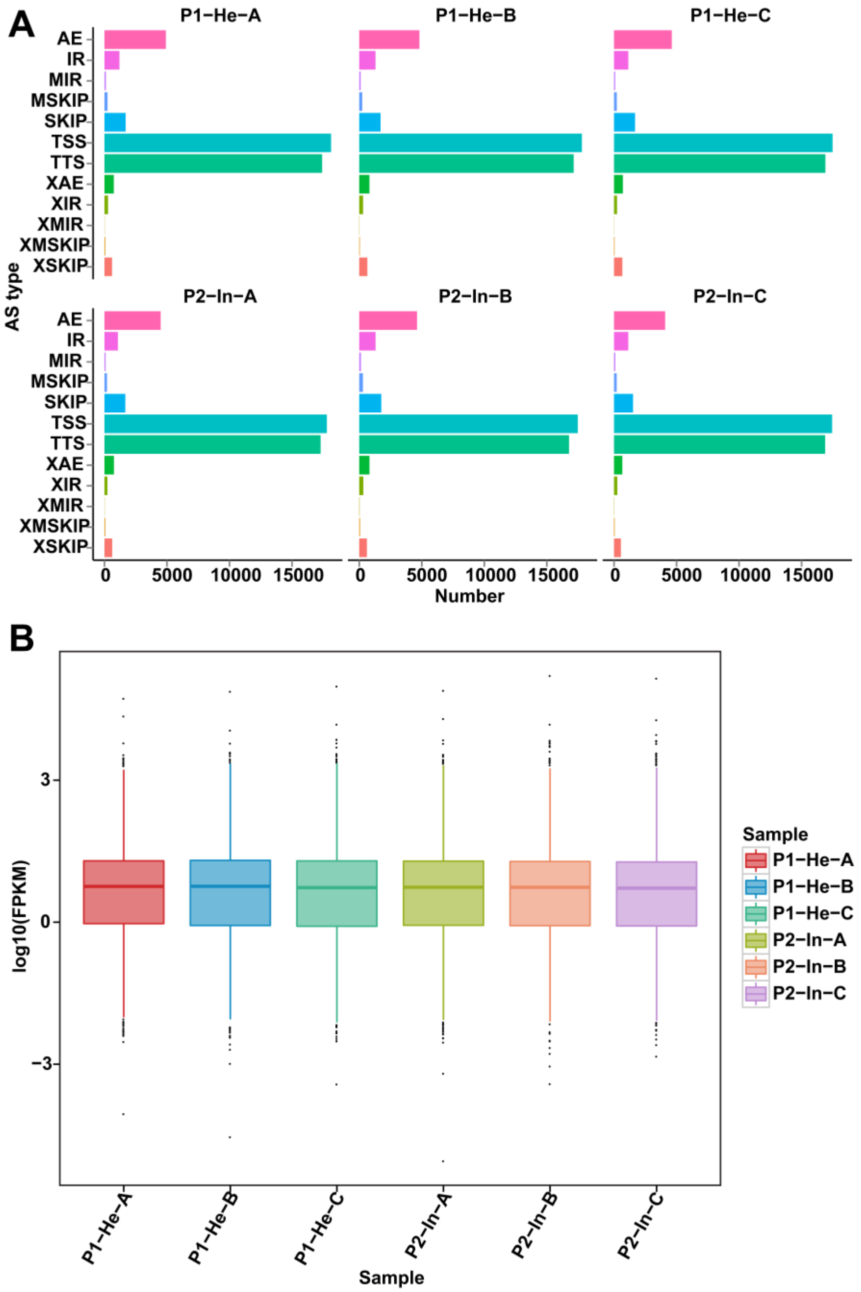

Supplementary Figure S8. Transcriptome analysis and differential gene expression in grapevine leaves in response to powdery mildew infection. All analyses were performed on RNA-Seq data from healthy control (P1-He, n=3) and powdery mildew-infected (P2-In, n=3) leaves of grapevine 'Yeniang No. 2'. (A) The distribution of different types of alternative splicing events detected across all samples. (B) Boxplots of FPKM values, confirming consistent expression distributions across samples.

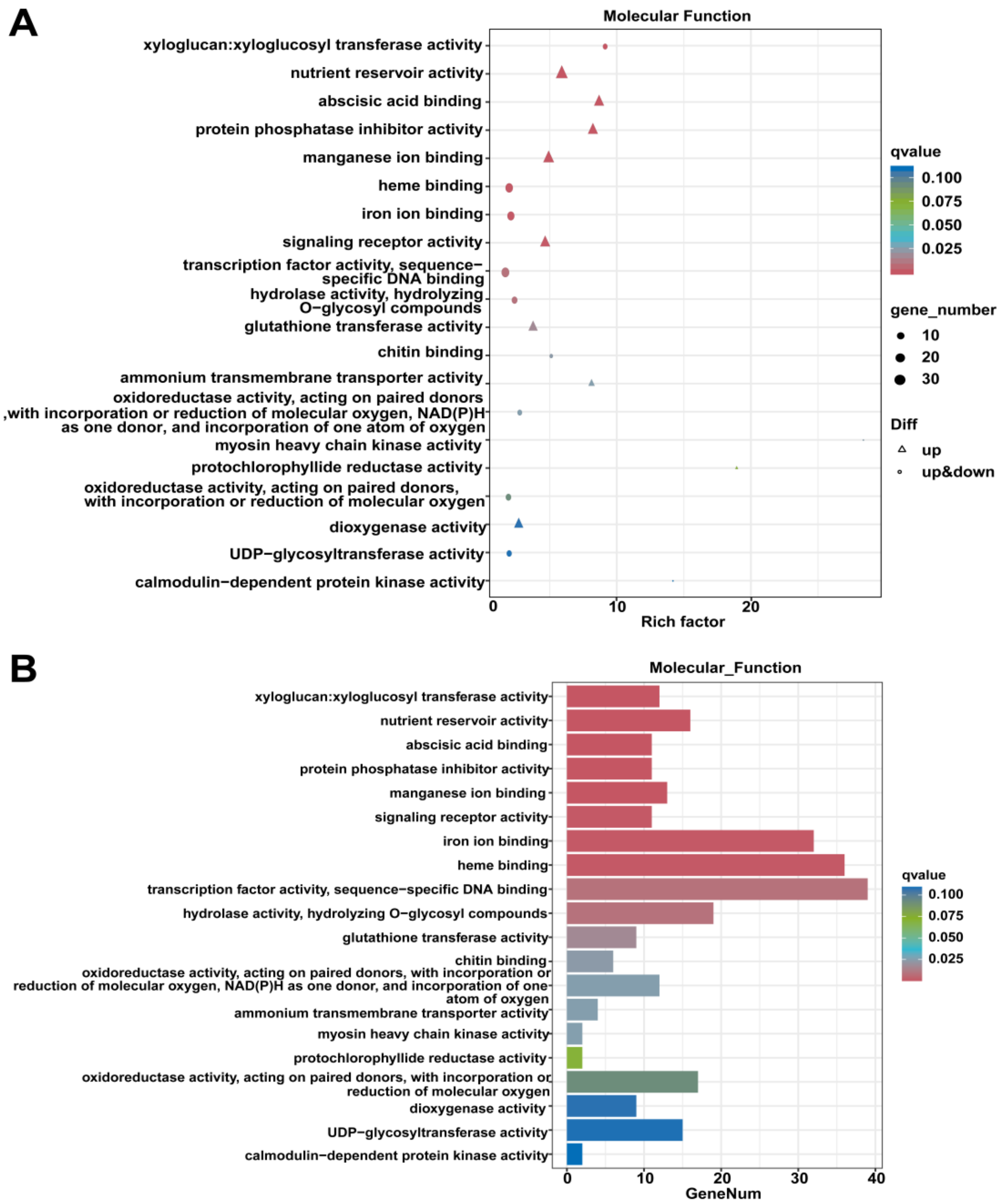

Supplementary Figure S9. Gene ontology (GO) enrichment analysis was conducted on the differentially expressed genes (DEGs). DEGs were identified by comparing healthy control (P1-He, n=3) and powdery mildew-infected (P2-In, n=3) grapevine 'Yeniang No. 2' leaves. Bubble plots of the top enriched GO terms for the (A) molecular function domain. Bar plots showing the number of DEGs in the most significantly enriched GO terms within the (B) molecular function domain.

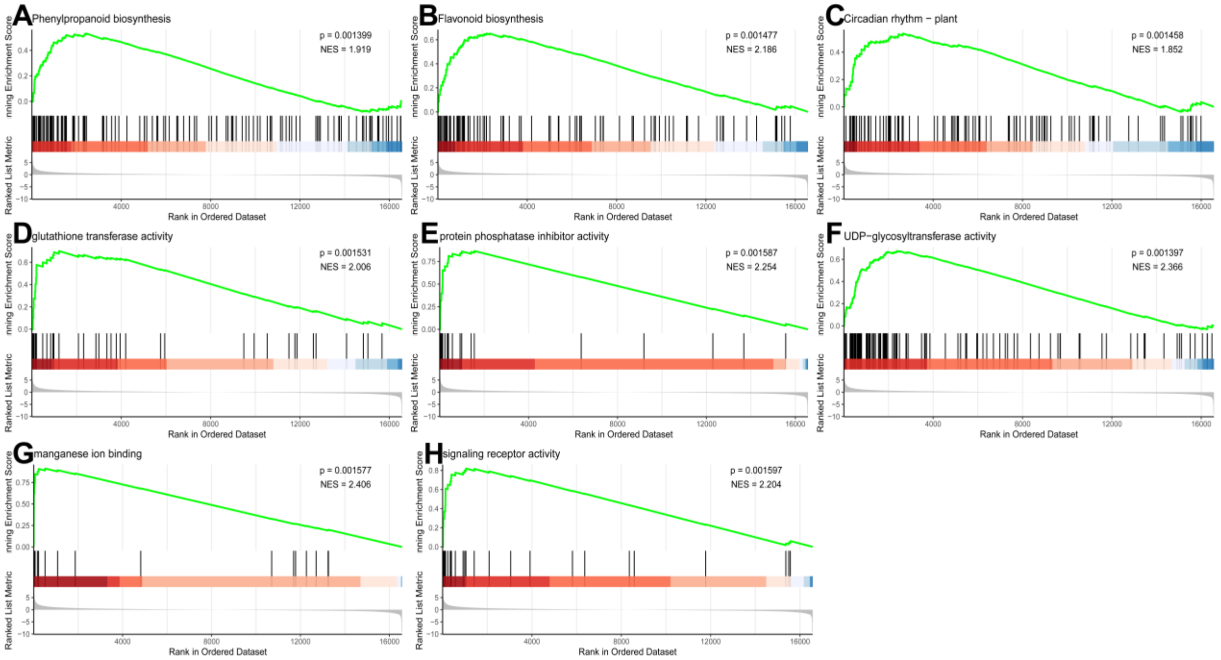

Supplementary Figure S10. The Gene Set Enrichment Analysis (GSEA) plot displays the KEGG pathway along with the molecular function domains of the Gene ontology (GO) gene set. The analysis compares gene expression profiles between healthy control (P1-He, n=3) and powdery mildew-infected (P2-In, n=3) grapevine 'Yeniang No. 2' leaves. The plot illustrates the distribution of a specific gene set across a list of all genes ranked by their log_2 fold change (log2FC). The top panel shows the running enrichment score (ES) as a green curve, with its peak representing the final ES for the set. The middle panel is a barcode plot where each vertical line indicates the position of a gene from the gene set within the ranked list. The bottom panel displays the log2FC values for all ranked genes, with red indicating positive and blue indicating negative correlation. A positive ES signifies enrichment of the gene set among up-regulated genes, while a negative ES signifies enrichment among down-regulated genes.

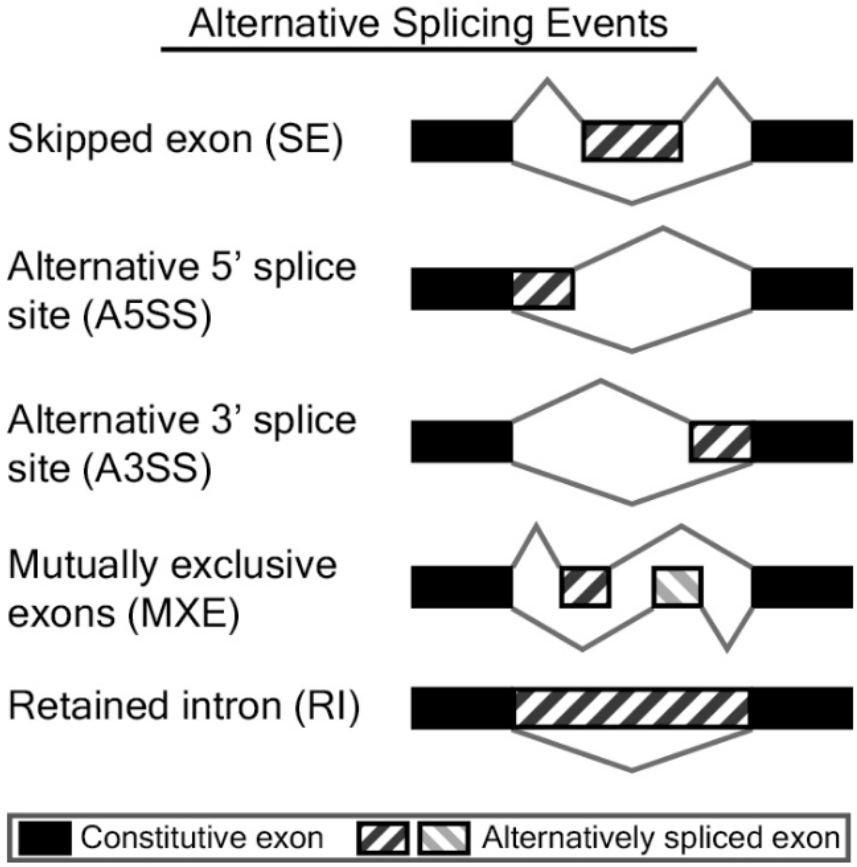

Supplementary Figure S11. Analysis of differential alternative splicing events between healthy and powdery mildew-infected grapevine leaves. The figure displays the distribution of the five major types of alternative splicing events identified in grapevine ‘Ye Niang 2’ using rMATS software. The analysis compares healthy control leaves with infected leaves (n=3 per condition). The event types were: skipped exon (SE), alternative 5' splice site (A5SS), alternative 3' splice site (A3SS), mutually exclusive exons (MXE), and retained intron (RI).

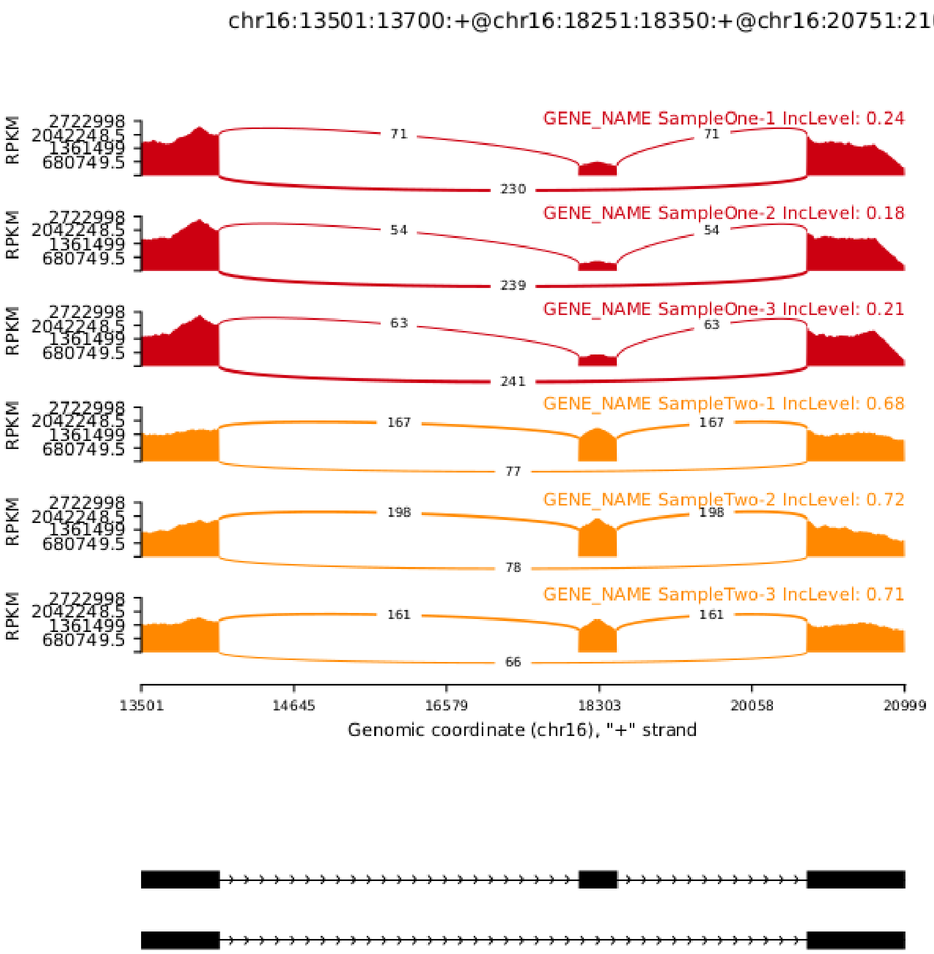

Supplementary Figure S12. Sashimi plot of a differential skipped exon (SE) event in grapevine 'Ye Niang 2'. The plot visualizes differences in splicing patterns between healthy control leaves (top three tracks) and leaves infected with powdery mildew (bottom three tracks). The bottom panel displays the gene model, where blocks represent exons and lines represent introns. Read coverage density across exons is shown as histograms for each sample. Arcs represent junction-spanning reads, with the number on each arc indicating the raw read count; the arc's thickness is proportional to this count. The calculated Inclusion Level (IncLevel), which represents the percentage of transcripts including the alternative exon, is provided for each sample.

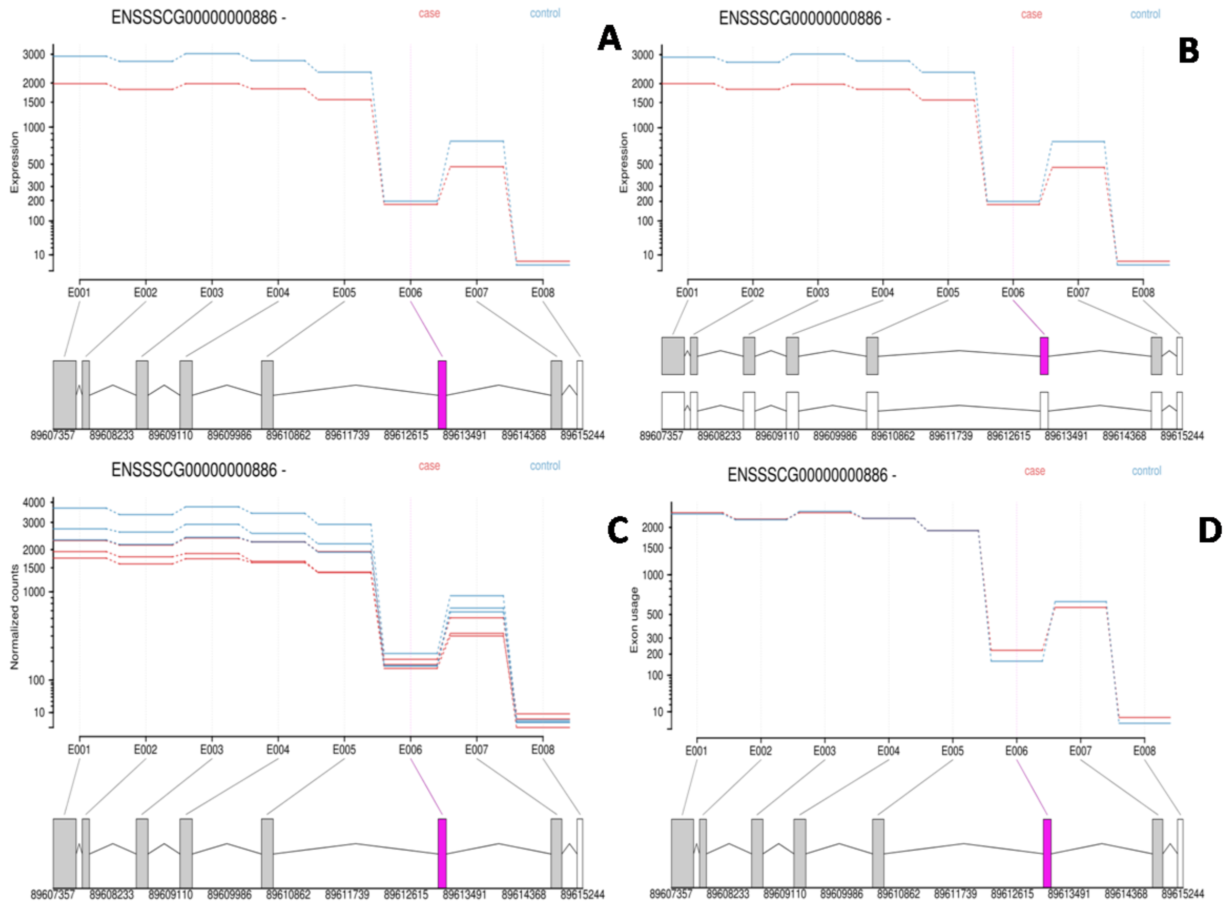

Supplementary Figure S13. Visualization of differential exon usage (DEU) for a representative gene in grapevine. The plot compares exon usage between healthy control leaves and leaves infected with powdery mildew from the grapevine cultivar ‘Ye Niang 2’. The panels display different aspects of the DEXSeq analysis: (A) Exon Expression: Estimated expression levels for each exon within the gene model. Red bars indicate exons with statistically significant differential usage between the two conditions. (B) Transcript Models: The known transcript isoforms of the gene, illustrating how the exons are combined. (C) Normalized Counts: The normalized read counts for each exon across all six samples, representing the raw data underlying the statistical model. (D) Splicing Coefficients: The relative exon usage, showing the effect of splicing changes while controlling for overall gene expression differences.

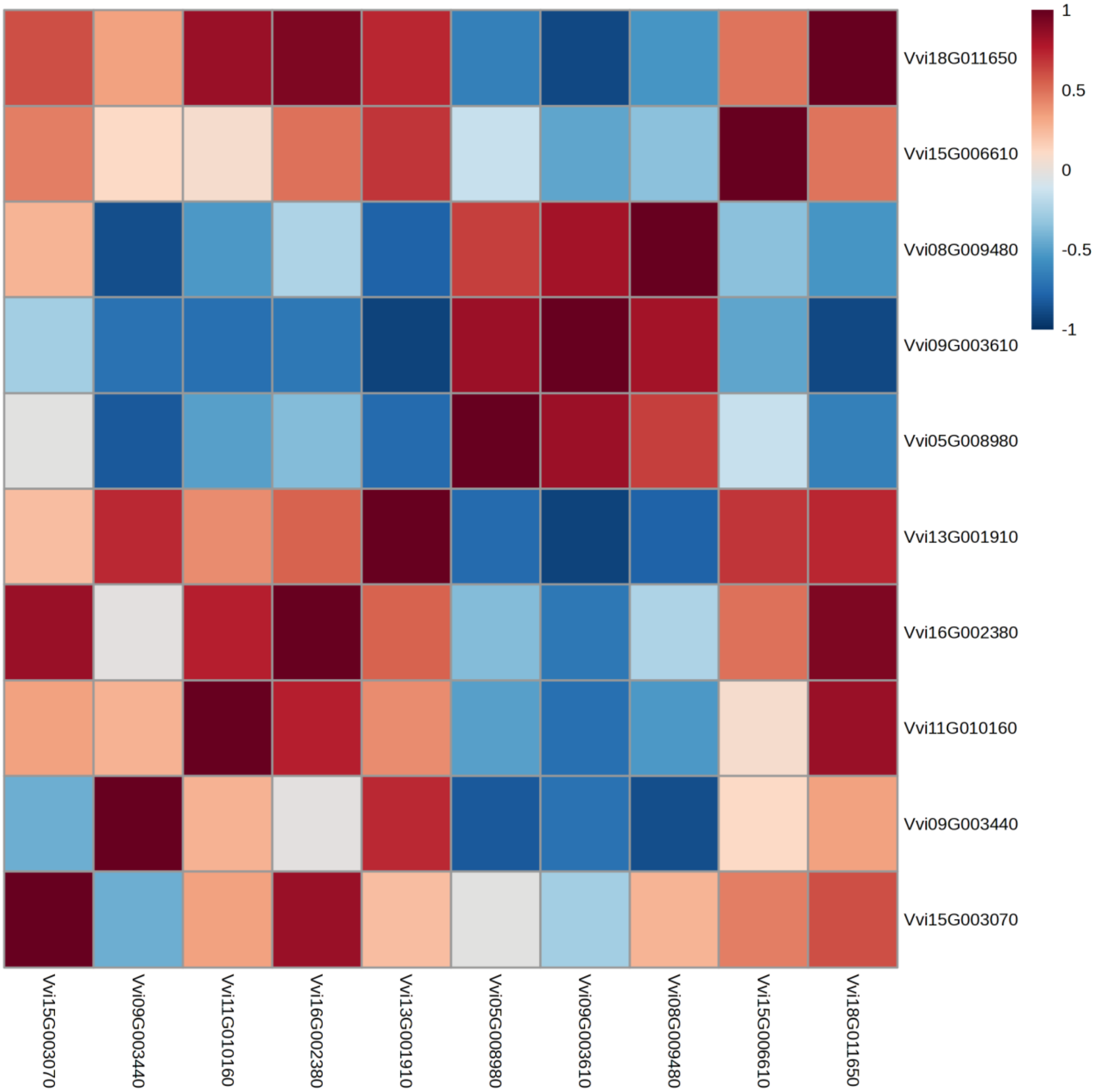

Supplementary Figure S14. Correlation heatmap of gene expression profiles for qPCR validation. This matrix displays the pairwise Pearson correlation coefficients for 10 candidate genes selected to validate transcriptome sequencing (RNA-Seq) data using quantitative real-time PCR (qPCR). The color scale represents the strength of the correlation, ranging from dark blue (strong negative correlation, -1) to dark red (strong positive correlation, +1). The hierarchical clustering reveals distinct groups of co-expressed genes, distinguishing between upregulated and downregulated patterns consistent with the transcriptomic analysis.
