# Supplementary material for: Transcriptome Profiling of Powdery Mildew-Stressed ‘Yeniang No. 2’ Grapevine Reveals Differential Expression, Alternative Splicing, and the Identification of 1232 Annotated Novel Genes
Source: Metabolites. 2026 Mar 9;16(3):182. doi: 10.3390/metabo16030182 (PMC13027967; doi:10.3390/metabo16030182)
Supplement: Supplementary file 1 [file metabolites-16-00182-s001.zip › Supplementary File S1/List of software and database.docx]

| **List of software included the version with main parameters** | | | |
| --- | --- | --- | --- |
| **Analysis of the content** | **Software Name** | **Version** | **Main parameters** |
| Genome comparison | HISAT2 | 2.0.4 | --dta -p 6 --max-intronlen 5000000 |
| Transcription splicing and merging | StringTie | v2.2.1 | --merge -F 0.1 -T 0.1 |
| Variability Separation Analysis | ASprofile | - | default |
| The transcripts obtained by StringTie are compared with the known gene group gff3 | gffcompare | 0.12.6 | default |
| Comparing Software in Functional Comments | Diamond | 2.0.15 | -k 100 -e -evalue 1e-5 -f 5 |
| GO Notes | InterProScan | 5.34-73.0 | -appl Pfam -goterms -iprlookup -pa -f xml -dp -t p |
| Pfam Comments in Feature Comments | hmmscan | 3.3.2 | --noali --cut_nc --acc --notextw |
| Difference Analysis Software | DESeq2 | 1.30.1 | default: test="Wald",fitType="parametric" |
| Difference Analysis Software | DESeq | 1.39.0 | default |
| Difference Analysis Software | edgeR | 3.32.1 | Agronomy: dispersion=0.01, EE: dispersion=0.16 |
| Difference Analysis Software | EBSeq | 1.30.0 | qtrm=0.5 |
| Aggregate analysis software | R/clusterProfiler | 4.4.4 | minGSSize=1,maxGSSize=10000,pAdjustMethod="fdr" |
| Aggregate analysis software | R/topGO | 2.48.0 | firstSigNodes=5 |
| GSEA Analysis Software | R/clusterProfiler | 4.4.4 | nPerm=1000 |
| Analysis of differential variable cut | rMATS | 4.0.2 | --cstat 0.0001 --anchorLength 1 |
| Differentiated exome analysis | DEXSeq | 1.12.2 | default |
| Transcription factor binding site prediction | R/TFBStools | 1.18.0 | min.score=90% |
| Analysis of transcription factor activity | R/CoRegNet | 1.2.0 | minTarg=10 |
| Fusion genetic analysis | FusionMap | 2015-01-09 | MinimalHit = 2,FusionReportCutoff = 1 |
| SNP/InDel | GATK | 3.2-2 | -dontUseSoftClippedBases -stand_call_conf 20.0 -stand_emit_conf 20.0 |
| SNP/InDel | SnpEff | 3.6c | default |

| **List of databases** | | |
| --- | --- | --- |
| **Database** | **Description** | **Homepage** |
| NR | non-redundant protein sequence database | <ftp://ftp.ncbi.nih.gov/blast/db/> |
| Swiss-Prot | A manually annotated, non-redundant protein sequence database | <http://www.uniprot.org/> |
| GO | Gene Ontology database | <http://www.geneontology.org/> |
| COG | The database of Clusters of Orthologous Groups of proteins | <http://www.ncbi.nlm.nih.gov/COG/> |
| KOG | The database of Clusters of Protein homology | <http://www.ncbi.nlm.nih.gov/KOG/> |
| Pfam | The database of Homologous protein family | <http://pfam.xfam.org/> |
| KEGG | The database of Kyoto Encyclopedia of Genes and Genomes | <http://www.genome.jp/kegg/> |
| STRING | Search Tool for the Retrieval of Interacting Genes/Proteins | <http://www.string-db.org/> |
| Ensembl | Database Sscrofa10.2 download from | <http://asia.ensembl.org/index.html> |
| Cosmic | COSMIC, is the world's largest and most comprehensive resource for exploring the impact of somatic mutations in human cancer. | <https://cancer.sanger.ac.uk/cosmic> |
| JASPAR | Database of transcription factor binding profiles | <http://jaspar.genereg.net/> |
