# Supplementary material for: Transcriptome Profiling of Powdery Mildew-Stressed ‘Yeniang No. 2’ Grapevine Reveals Differential Expression, Alternative Splicing, and the Identification of 1232 Annotated Novel Genes
Source: Metabolites. 2026 Mar 9;16(3):182. doi: 10.3390/metabo16030182 (PMC13027967; doi:10.3390/metabo16030182)
Supplement: Supplementary file 1 [file metabolites-16-00182-s001.zip › Supplementary Tables.pdf]

Supplementary Table S1. Primer pairs for qPCR analysis of 10 DEGs

Gene ID	Primers sequences
Vvi09G003610	F: CATGCCTGTGAAGATGCAGATC
	R: CGAAGAAGAGGCCCCTTTGA
Vvi13G001910	F: GGACCCTCACAAAACATCCTTC
	R: AGAAGTCTCTCAGGCATCACTT
Vvi08G009480	F: CTCGTTTATTTTCGTCTGGGCT
	R: CAAATCATCGGCCTCTCCTTTT
Vvi18G011650	F: GTTGCTGGGTTTGAATGGAAAC
	R: TTTGCACAAATCCCGAGGTATC
Vvi05G008980	F: GCAAGCCCAAATCTGAGAAAGA
	R: GAATATGAACCCCTTGCGAACA
Vvi15G003070	F: GAAGAAGATCACACATGCACCA
	R: TCCACCATGAATTGCTTCCATC
Vvi11G010160	F: TGGAACCGAATCTCAGGTAACA
	R: TGATCCACTTCACGTACCAGAT
Vvi15G006610	F: GTGAATGGGAACGGTAAAGGAG
	R: GCTGTTGTCATCTTCGCTTTTG
Vvi09G003440	F: CTGCATCAACAAAAACCAGCTC
	R: GAAAACCCATTTTCCAGACCCA
Vvi16G002380	F: TCCAGACCATTCTTCCTCCAAA
	R: CTGATACTGTGTCTCTGCCTCT
VvActin	F: TCCTTGCCTTGCGTCATCTAT
	R: CACCAATCACTCTCCTGCTACAA

Supplementary Table S2. Evaluating the quality and summarizing RNA-sequencing libraries. The table shows the sequencing output and quality metrics for six libraries made from leaves of the grapevine 'Ye Niang 2.' There were two groups of leaves in the experiment: healthy control leaves (P1-He-A, P1-He-B, P1-He-C; n=3) and leaves that had been infected with powdery mildew (P2-In-A, P2-In-B, P2-In-C; n=3).

Sample name	Clean reads	Clean bases	GC Content	%≥Q30
P1-He-A	21,124,276	6,327,635,250	46.46%	92.86%
P1-He-B	20,551,515	6,154,167,368	45.88%	92.58%
P1-He-C	20,157,791	6,034,921,606	46.40%	92.24%
P2-In-A	21,038,597	6,299,806,218	46.55%	92.64%
P2-In-B	19,658,063	5,885,921,112	46.21%	92.62%
P2-In-C	20,512,792	6,143,285,420	46.48%	93.46%

Note: Clean reads: the total number of pair-end reads in the clean data; Clean bases: total base number of Clean Data; GC content: Percentage of G,C in clean data; ≥Q30%: Percentage of bases with Q-score no less than Q30.

Supplementary Table S3. Alignment statistics of RNA-sequencing reads to the grapevine reference genome. The table presents mapping results for libraries from healthy control (P1-He) and powdery mildew-infected (P2-In) leaves of the grapevine cultivar 'Ye Niang 2'. Results for three biological replicates per condition are shown (indicated by suffixes -A, -B, and -C).

Sample name	Total Reads	Mapped Reads	Uniq Mapped Reads	Multiple Map Reads	Reads Map to '+'	Reads Map to '-'
P1-He-A	42,248,552	37,881,461 (89.66%)	36,068,748 (85.37%)	1,812,713 (4.29%)	20,397,917 (48.28%)	20,440,786 (48.38%)
P1-He-B	41,103,030	36,651,966 (89.17%)	35,032,174 (85.23%)	1,619,792 (3.94%)	19,622,031 (47.74%)	19,678,464 (47.88%)
P1-He-C	40,315,582	35,810,722 (88.83%)	34,319,345 (85.13%)	1,491,377 (3.70%)	19,088,221 (47.35%)	19,146,965 (47.49%)
P2-In-A	42,077,194	37,396,858 (88.88%)	35,373,279 (84.07%)	2,023,579 (4.81%)	20,412,964 (48.51%)	20,456,430 (48.62%)
P2-In-B	39,316,126	34,901,294 (88.77%)	33,220,741 (84.50%)	1,680,553 (4.27%)	18,822,375 (47.87%)	18,889,446 (48.05%)

Note: Total Reads: Counts of Clean Reads, counted as single end; Mapped Reads: Counts of mapped reads and the proportion of that in clean data; Uniq Mapped Reads: Counts of reads mapped to a unique position on reference genome and proportion of that in clean data; Multiple Mapped Reads: Counts of reads mapped to multiple positions on reference genome and proportion of that in clean data; Reads Map to '+': Counts of reads mapped to the sense chain and the proportion of that in clean data; Reads Map to '-': Counts of reads mapped to antisense chain and proportion of that in clean data.

Supplementary Table S4. Summary of alternative splicing events in healthy and infected grapevine leaves. The table displays the counts for major alternative splicing types identified in healthy control (P1-He; n=3) and powdery mildew-infected (P2-In; n=3) leaves of the grapevine ‘Ye Niang 2’.

**AS**	**P1-He-A**	**P1-He-B**	**P1-He-C**	**P2-In-A**	**P2-In-B**	**P2-In-C**
XSKIP	604	647	665	618	611	546
XMSKIP	61	46	43	60	58	52
XMIR	16	18	11	15	30	19
XIR	284	305	239	237	322	261
XAE	745	815	706	754	820	661
TTS	17405	17145	16898	17283	16776	16883
TSS	18120	17805	17477	17785	17474	17442
SKIP	1693	1704	1679	1667	1764	1518
MSKIP	245	238	221	204	291	213
MIR	113	114	94	93	134	104
IR	1195	1300	1141	1075	1303	1137
AE	4908	4808	4619	4489	4619	4085

Note: TSS: Alternative 5' first exon (transcription start site) the first exon splicing; TTS: Alternative 3' last exon (transcription terminal site) the last exon splicing; SKIP: Skipped exon(SKIP_ON,SKIP_OFF pair) single exon skipping; XSKIP: Approximate SKIP (XSKIP_ON,XSKIP_OFF pair) single exon skipping (fuzzy boundary); MSKIP: Multi-exon SKIP (MSKIP_ON,MSKIP_OFF pair) multi-exon skipping; XMSKIP: Approximate MSKIP (XMSKIP_ON,XMSKIP_OFF pair) multi-exon skipping (fuzzy boundary); IR: Intron retention (IR_ON, IR_OFF pair) single intron retention; XIR: Approximate IR (XIR_ON,XIR_OFF pair) single intron retention (fuzzy boundary); MIR: Multi-IR (MIR_ON, MIR_OFF pair) multi-intron retention; XMIR: Approximate MIR (XMIR_ON, XMIR_OFF pair) multi-intron retention (fuzzy boundary); AE: Alternative exon ends (5', 3', or both); XAE: Approximate AE variable 5' or 3' end (fuzzy boundary);

Supplementary Table S5. Classification and quantification of differential alternative splicing (DAS) events in response to powdery mildew infection. The table shows the number of significant DAS events identified between healthy control (P1-He; n=3) and powdery mildew-infected (P2-In; n=3) leaf samples from the grapevine cultivar 'Ye Niang 2'.

DEG Set	A3SS	A5SS	MXE	RI	SE
P1-He_vs_P2-In	1,582	1,169	1,893	2,427	15,139

Note: DEG set: Name of DEG set; The rest columns: number of DEGs in corresponding alternative splicing events; A3SS: Alternative 3' splice junction; A5SS: Alternative 5' splice junction; MXE: Mutually exclusive exons; RI: Intron retention; SE: Exon skipping.
