# Supplementary figures and images for: Transcriptome Profiling of Powdery Mildew-Stressed ‘Yeniang No. 2’ Grapevine Reveals Differential Expression, Alternative Splicing, and the Identification of 1232 Annotated Novel Genes
Source: Metabolites. 2026 Mar 9;16(3):182. doi: 10.3390/metabo16030182 (PMC13027967; doi:10.3390/metabo16030182)

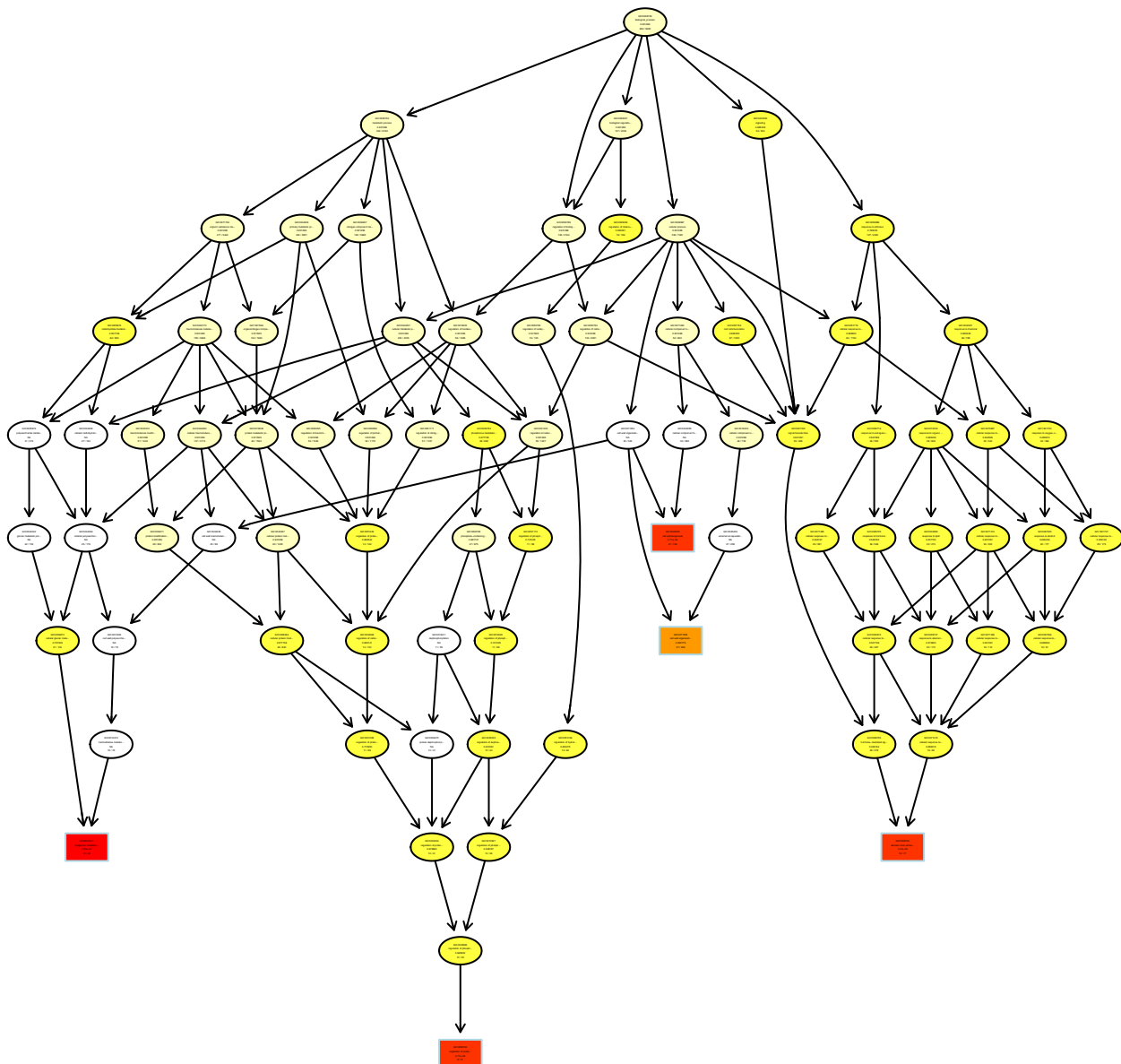

Supplement: Supplementary file 1 [file metabolites-16-00182-s001.zip › Supplementary File S15/All/P1-He_vs_P2-In.topGO_BP.pdf]

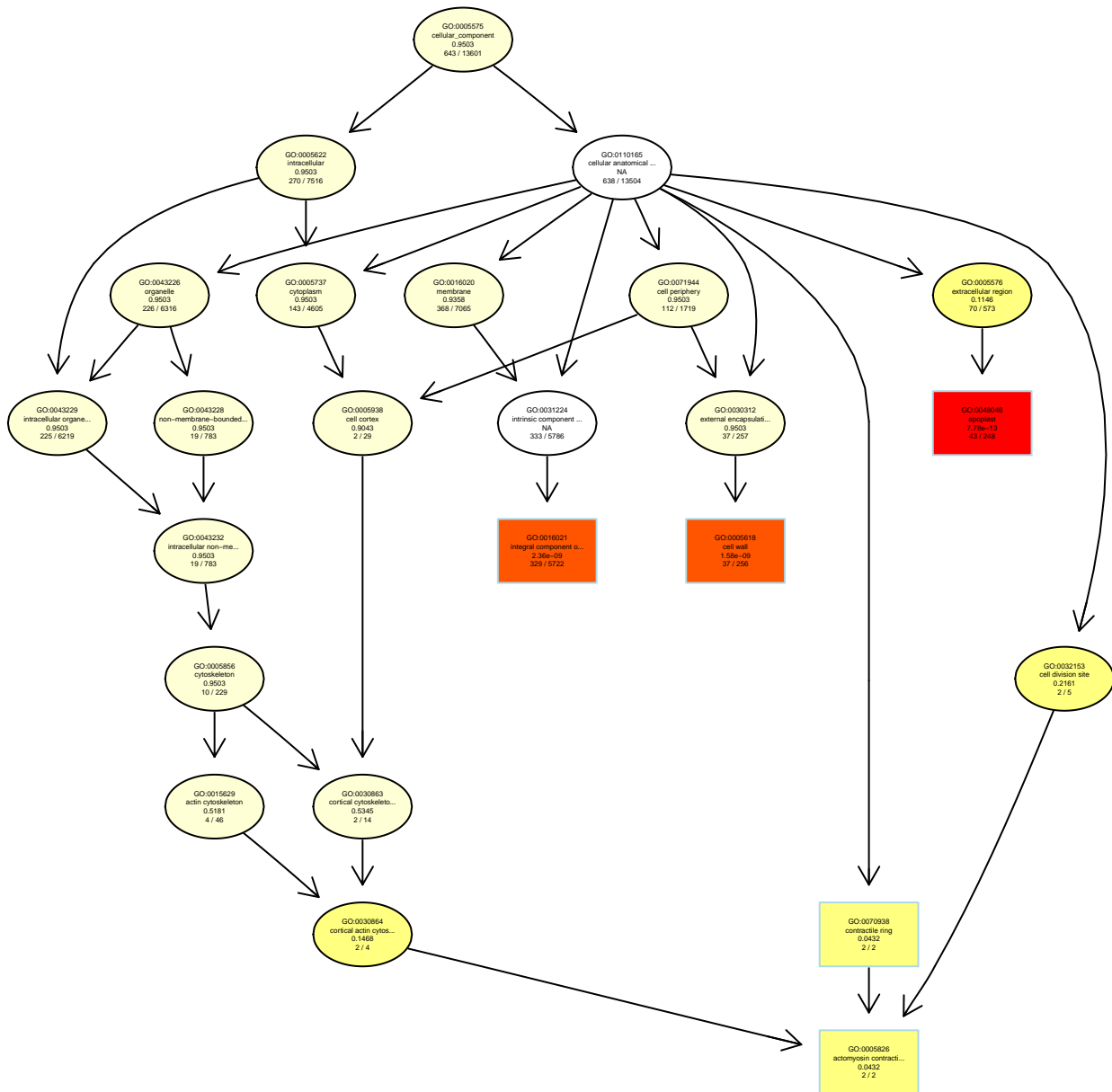

Supplement: Supplementary file 1 [file metabolites-16-00182-s001.zip › Supplementary File S15/All/P1-He_vs_P2-In.topGO_CC.pdf]

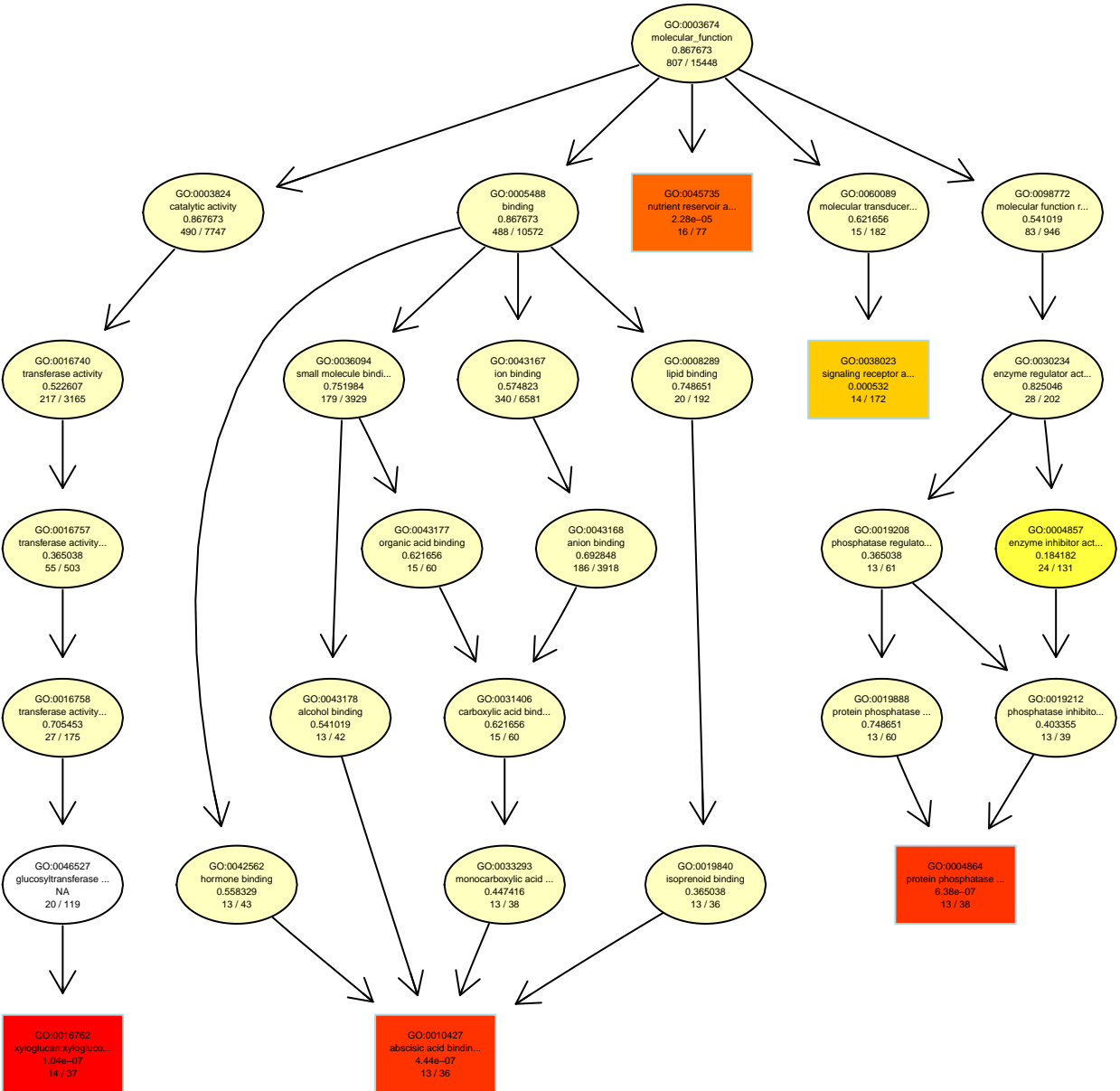

Supplement: Supplementary file 1 [file metabolites-16-00182-s001.zip › Supplementary File S15/All/P1-He_vs_P2-In.topGO_MF.pdf]

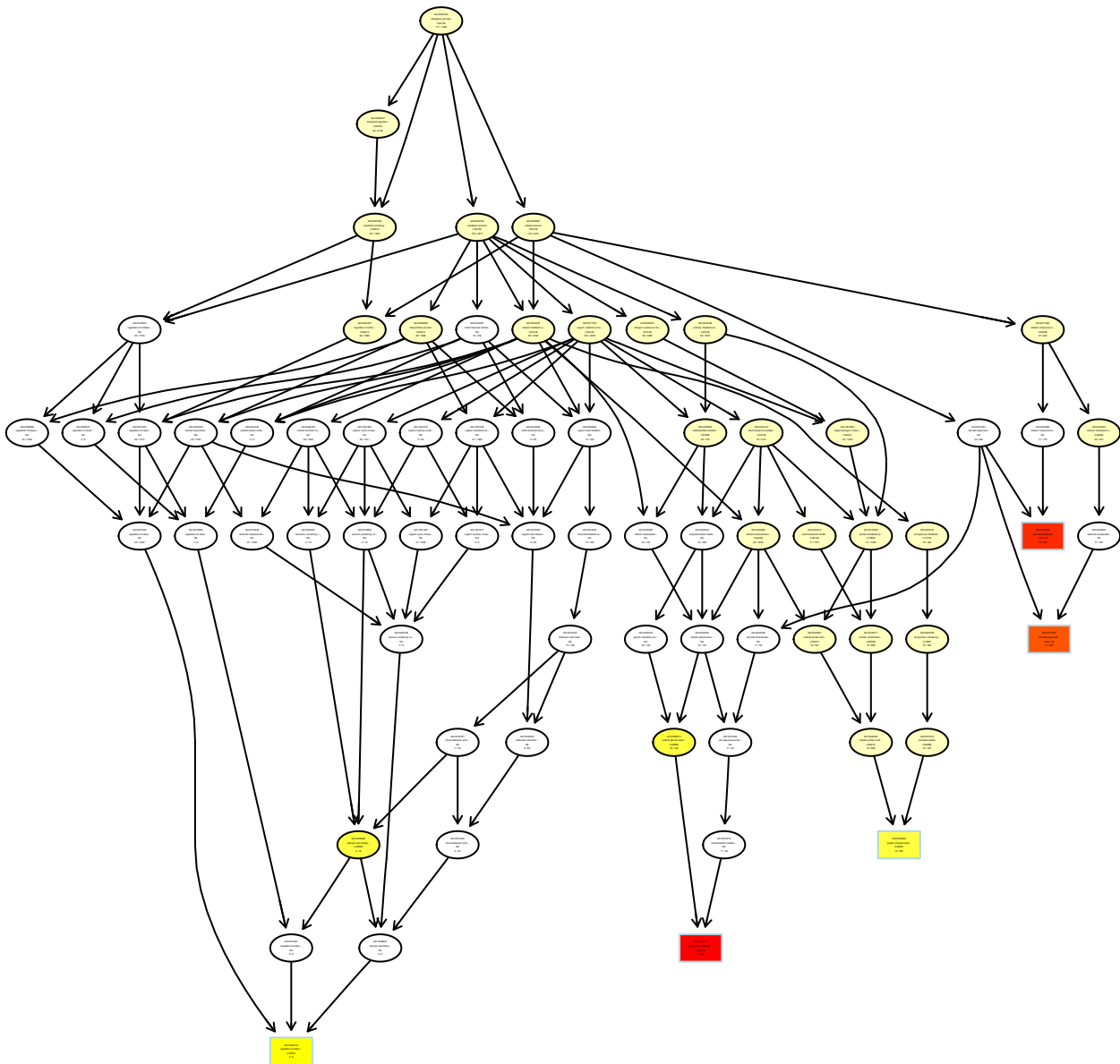

Supplement: Supplementary file 1 [file metabolites-16-00182-s001.zip › Supplementary File S15/down/P1-He_vs_P2-In.topGO_BP.pdf]

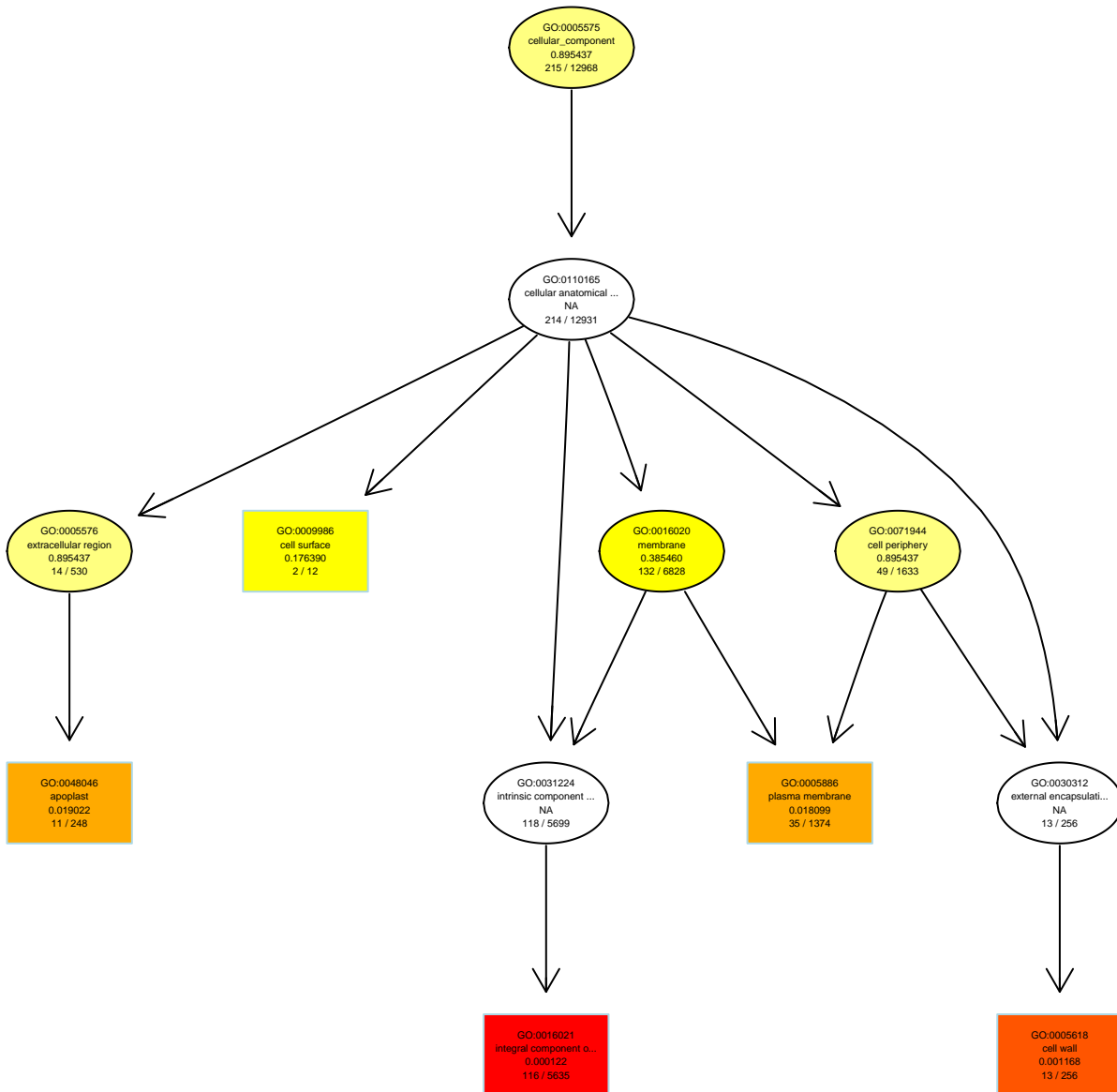

Supplement: Supplementary file 1 [file metabolites-16-00182-s001.zip › Supplementary File S15/down/P1-He_vs_P2-In.topGO_CC.pdf]

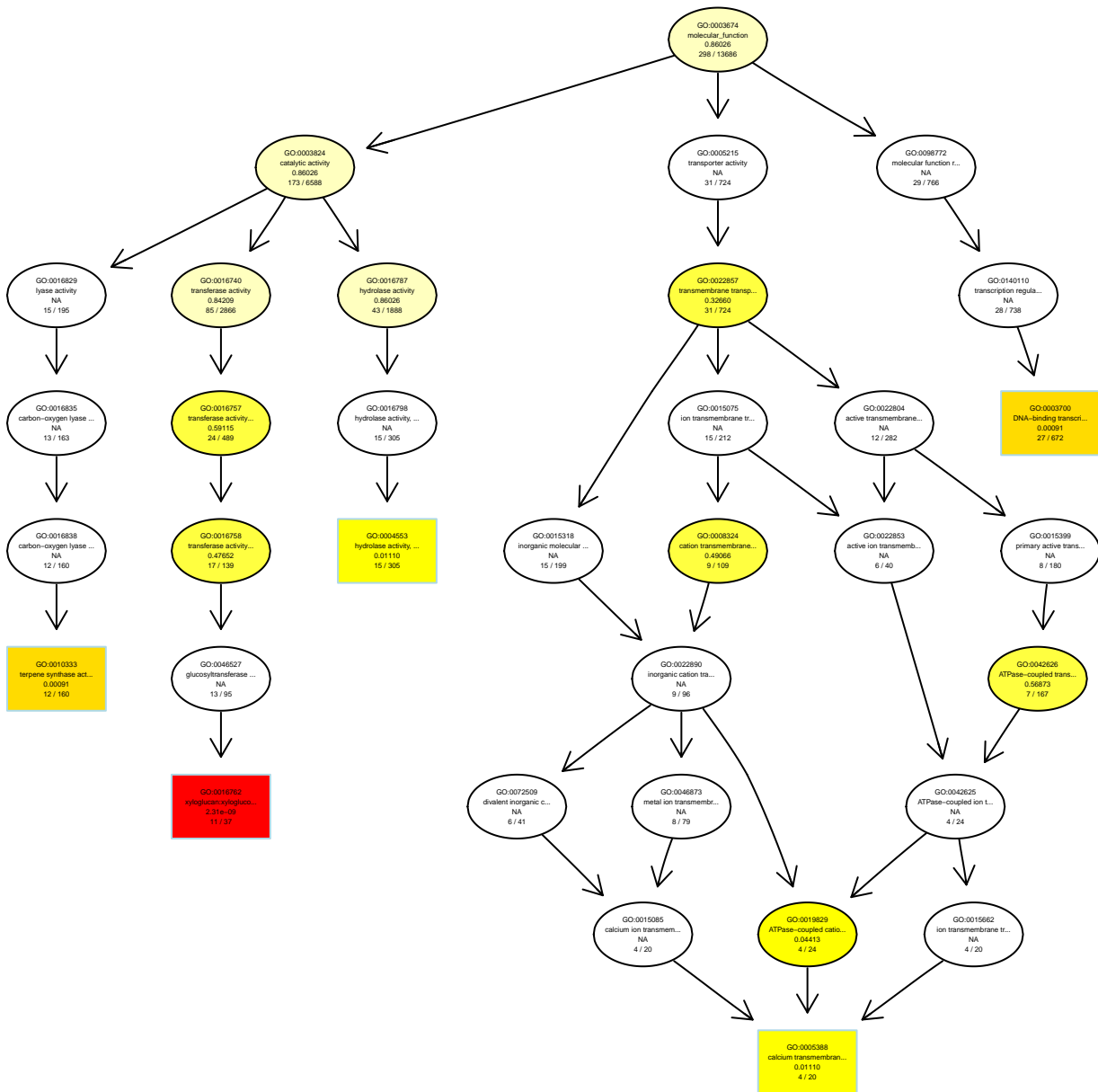

Supplement: Supplementary file 1 [file metabolites-16-00182-s001.zip › Supplementary File S15/down/P1-He_vs_P2-In.topGO_MF.pdf]

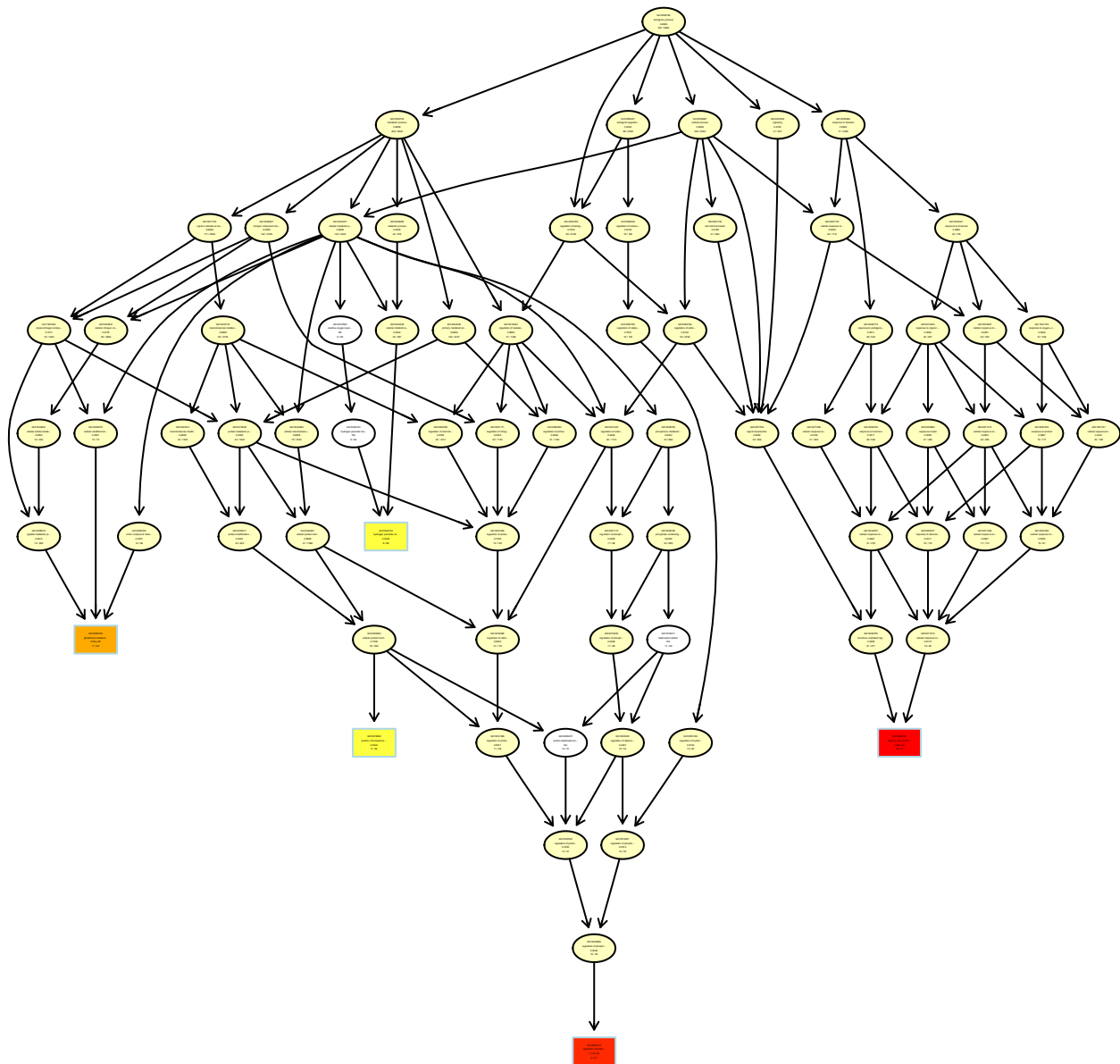

Supplement: Supplementary file 1 [file metabolites-16-00182-s001.zip › Supplementary File S15/up/P1-He_vs_P2-In.topGO_BP.pdf]

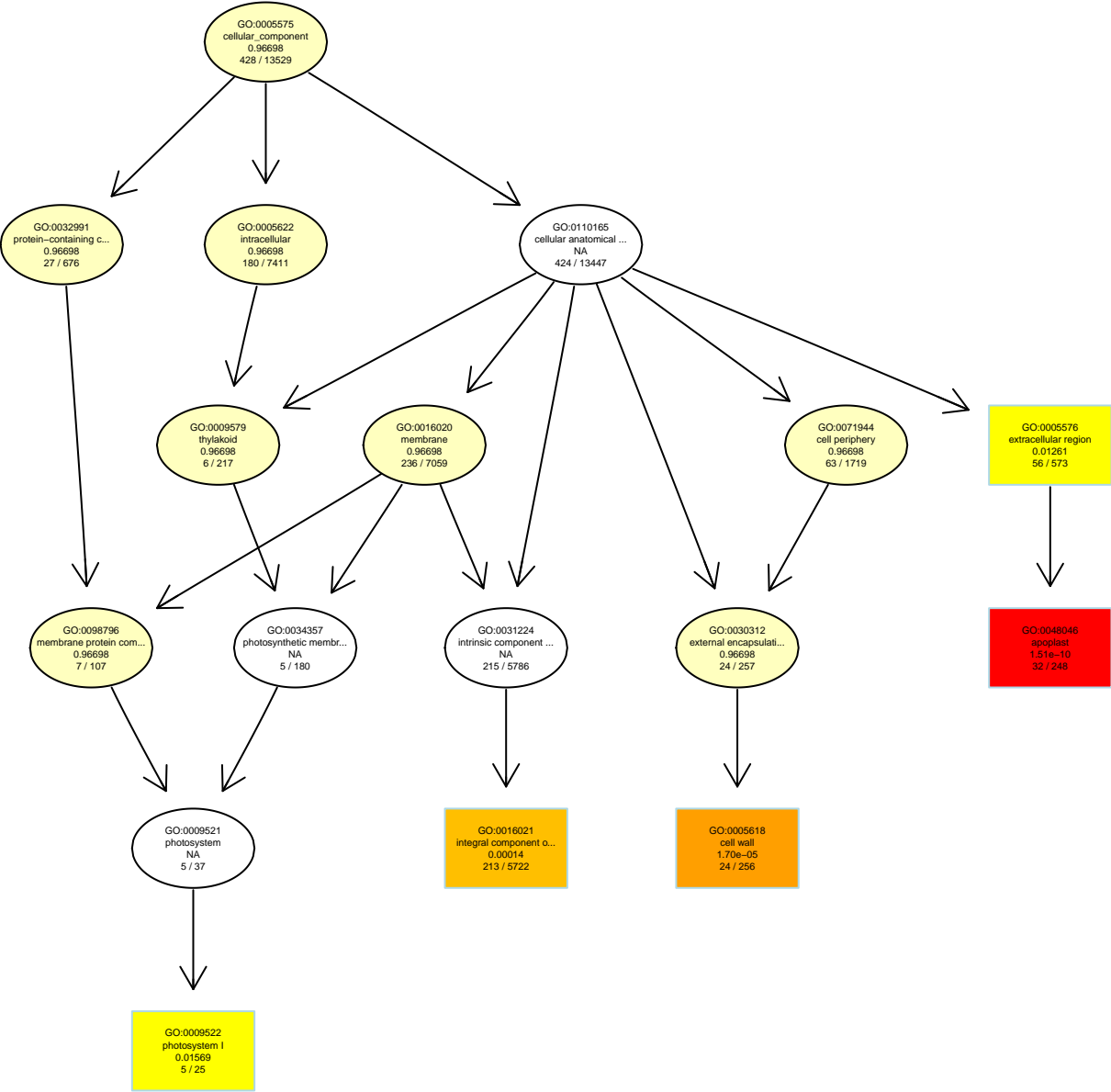

Supplement: Supplementary file 1 [file metabolites-16-00182-s001.zip › Supplementary File S15/up/P1-He_vs_P2-In.topGO_CC.pdf]

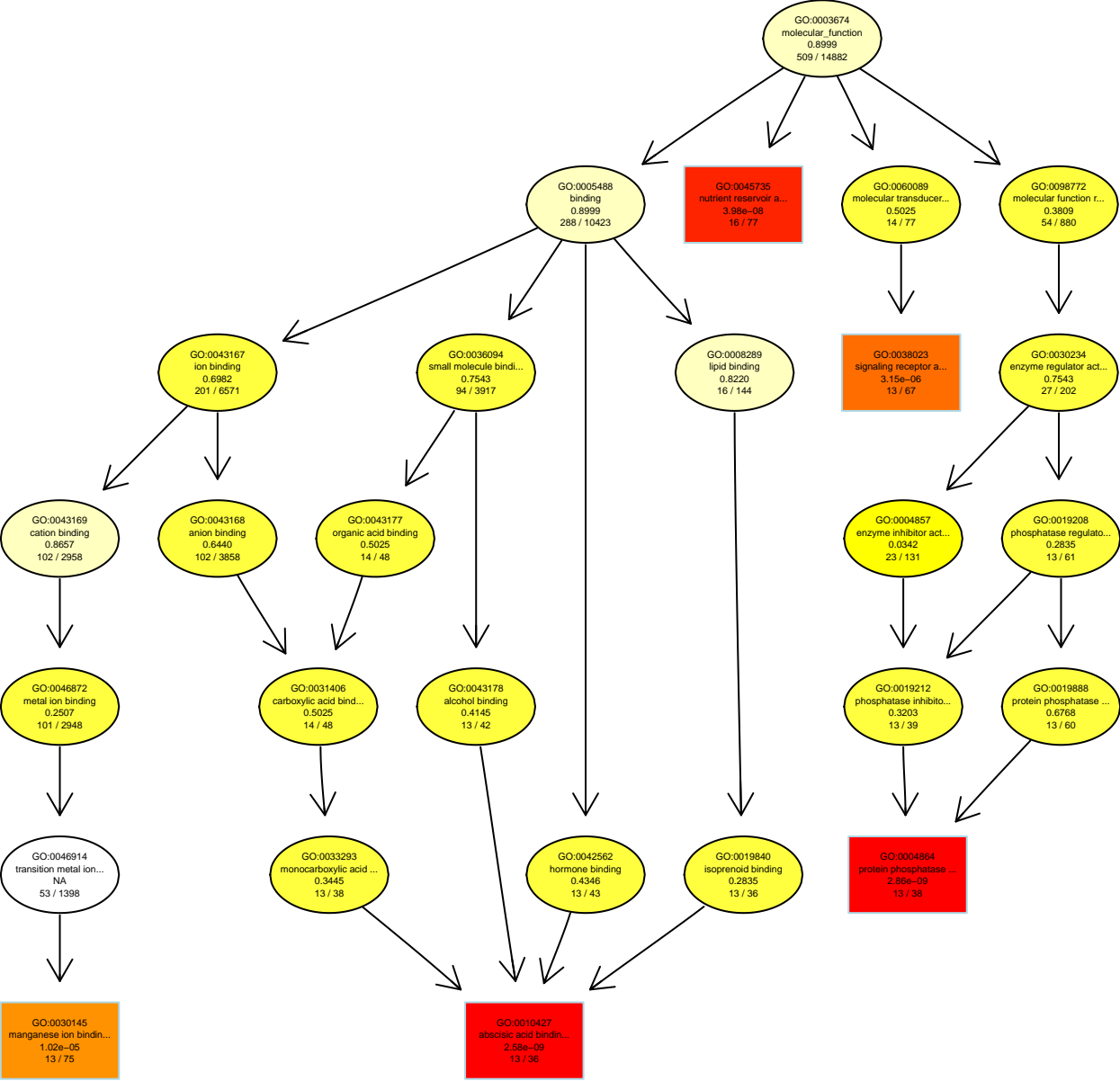

Supplement: Supplementary file 1 [file metabolites-16-00182-s001.zip › Supplementary File S15/up/P1-He_vs_P2-In.topGO_MF.pdf]
